# Properties of peptides released from salmon and carp *via* simulated human-like gastrointestinal digestion described applying quantitative parameters

**DOI:** 10.1371/journal.pone.0255969

**Published:** 2021-08-10

**Authors:** Justyna Borawska-Dziadkiewicz, Małgorzata Darewicz, Anna Sylwia Tarczyńska

**Affiliations:** 1 Faculty of Food Science, Department of Food Biochemistry, University of Warmia and Mazury in Olsztyn, Olsztyn, Poland; 2 Faculty of Food Science, Department of Dairy Science and Quality Management, University of Warmia and Mazury in Olsztyn, Olsztyn, Poland; Escola Paulista de Medicina, BRAZIL

## Abstract

Apart from the classical (experimental) methods, biologically active peptides can be studied *via* bioinformatics approach, also known as *in silico* analysis. This study aimed to verify the following research hypothesis: ACE inhibitors and antioxidant peptides can be released from salmon and carp proteins during simulated *in silico* human-like gastrointestinal digestion. The potential to release biopeptides was evaluated using the BIOPEP-UWM quantitative criteria including the profile of biological activity, frequency of the occurrence (A)/release (A_E_) of fragments with an ACE inhibitory or antioxidant activity by selected enzymes, and relative frequency of release of bioactive fragments with a given activity by selected enzymes (W). Salmon collagen and myofibrillar proteins of carp turned out to be the best potential source of the searched peptides–ACE inhibitors and antioxidant peptides. Nonetheless, after digestion, the highest numbers of ACE inhibitors and antioxidant peptides were potentially released from the myofibrillar proteins of salmon and carp. Peptide Ranker Score, Pepsite2, and ADMETlab platform were applied to evaluate peptides’ bioactivity potential, their safety and drug-like properties. Among the 63 sequences obtained after the simulated digestion of salmon and carp proteins, 30 were considered potential biopeptides. The amino acid sequences of ACE-inhibiting and antioxidant peptides were predominated by P, G, F, W, R, and L. The predicted high probability of absorption of most analyzed peptides and their low toxicity should be considered as their advantage.

## Introduction

In the last decade, an unprecedented increase has been observed in the number of research addressing food products as the natural source of components potentially useful in preventing diet-related non-communicable diseases (DNCD), offering an alternative to pharmacological treatment. Because the unhealthy diet is one of the main lifestyle-related risk factors in DNCD, the World Health Organization (WHO) has developed a global strategy for DNCD prevention and control that entails a well-balanced diet based on, among other things, functional foods [1]. Among the variety of functional food ingredients, particular attention has been paid to biologically active peptides (BPs). Their consumption has been reported to offer multiple health benefits, while their use for prophylactic and therapeutic purposes has been investigated by many research centers worldwide [2,3]. Many scientific reports have emphasized the role of bioactive, usually small (2–20 amino acids long), peptides derived from food proteins. After enzymatic digestion, these peptides are released from the intact proteins and enter into reactions with body receptors to regulate body functions [4]. Ample research works have indicated hydrolysates and/or peptides of food proteins to inhibit angiotensin I-converting enzyme (ACE) [EC 3.4.15.1] and show antioxidant activity. These include protein hydrolysates of, among others, milk, wheat, soybean, egg, shrimp, and fish, like capelin, mackerel, herring, tuna, common sole, or Alaska pollock [5,6].

Many ACE inhibitors have been discovered over the years, including synthetic compounds and peptides derived from food protein hydrolysates [7]. Today, ACE inhibitors are the preferred first-line therapy for hypertension (for example, synthetic ACE inhibitor—Captopril), and interest in them continues to increase [7,8]. Natural ACE-inhibiting peptides do not cause many side effects, such as rash, swelling, impaired kidney function, which are characteristic of synthetic drugs, but at the same time, they are not as active as the latter.

Reactive oxygen species (ROS) in the forms of superoxide anion (·O_2_^−^), hydroxyl radical (·OH), and hydrogen peroxide (H_2_O_2_) are metabolites likely to occur in the natural environment. Because they are involved in oxidation reactions, they cause damage to nucleic acids (e.g., modification of their composition and configuration), lipids (e.g., peroxidation of plasma lipoproteins), fatty acids (e.g., peroxidation of fatty acids in cell membranes), and proteins (e.g., modification of the composition and configuration of amino acid residues). Consequently, free radicals can be one of the etiological factors of many lifestyle-related diseases, including cardiovascular diseases [9].

According to the scientific reports, fish proteins can be a valuable source of health-promoting peptides, i.e. ACE inhibitory and antioxidant peptides. The main farmed fish species in Europe include carp (*Cyprinus carpio*) in Central and East Europe and Atlantic salmon (*Salmo salar*) in Scandinavia. In terms of the economic value, salmon is among the most important fish market products in the European Union (EU). What is more, Poland, where imported salmon is filleted and smoked or frozen, is one of its largest exporters in the EU [10,11].

Carp and salmon feature growth capability, resistance to adverse environmental conditions, diseases, and parasites, as well as adaptability and regeneration potential. The breeding success was also determined by their taste and nutritional values, including a relatively high protein content (16–20%) with a beneficial amino acid composition and a high digestibility (over 98%) [12]. When included in an everyday diet, salmon and carp proteins could become the source of peptides with ACE-inhibitory and antioxidant properties [13,14] to be used in DCND prevention.

Laboratory screening of BPs is a challenging, costly, and time-consuming process. In turn, computational methods can offer an inexpensive, time-saving but effective means of predicting the presence, profiling, and selecting BPs from food protein hydrolysates [15,16]. Information on bioactive proteins and peptides, as well as other food ingredients, is available in online databases [16,17]. Chemical compound databases are popular and widely used tools in biological and chemical sciences and are becoming increasingly popular in food science [18]. Bioinformatic tools available in databases allow simulating protein hydrolysis. During *in silico* simulated human-like gastrointestinal digestion, specific enzymes are selected that can release bioactive fragments from intact proteins [17]. Independently, some virtual screening methods are used to look for peptide inhibitors that can be expanded into food drug-like compounds. Such an approach is called target/structure-based prediction/identification, meaning the exploration of compound-target interactions. Additionally, the knowledge of undesirable properties of the obtained hydrolysates (e.g., toxicity, allergenicity) is useful considering their use as a component of functional food or dietary supplements [19,20]. To summarize, this study aimed to verify the research hypothesis assuming that salmon and carp proteins can be potential sources of peptides featuring ACE inhibitory activity and/or antioxidant activity, and that these peptides can be released during *in silico* hydrolysis simulating digestion in the human body.

## Materials and methods

### Materials

*In silico* studies were carried out with selected amino acid sequences of proteins of salmon (*Salmo salar*) and carp (*Cyprinus carpio*) available in the UniProt database of protein sequences (providers: Swiss Institute of Bioinformatics, Switzerland and European Bioinformatics Institute, UK; https://www.uniprot.org/) (accessed: September 2020) [21]. Amino acid were divided into three groups: myofibrillar (myosin and actin), sarcoplasmic (myoglobin and parvalbumin), and other proteins including collagen (S1 Table). Identical sequences were eliminated using the Clustal Omega ‒ Multiple Sequence Alignment program with default settings [13,14,22]. In result, total of 85 amino acid sequences of salmon (52) and carp (33) proteins with less than 90% identity were selected for further analyses. They represented salmon myofibrillar proteins (UniProt ID: Q78BU2, B5XFZ3, B5DG40, B5DH12, B5DH13, B5DGT2, B9EMP7, B5XFD6, B5XFN3, Q7ZZN0, B5X1K8, B9ENW2, B5XE45, B5X5V3, B5DGT1, A8WCK1, C0PU27, B5XCI5, C0PU50, B9ELW1, C0PU83, C0PUQ9, C0PUQ3, Q2HXU3), salmon sarcoplasmic proteins (B5DGI8, C0HAT9, Q91482, Q91483, B5X6D1, B9ENR7, B9EPT7, B5X603, B9ENY2), other salmon proteins (C0H9S7, B5X659, C1K2L7, A7KE05, C0H805, B5X746, B5X8L0, C0H744, P21848, C0HBF1, B5X4Z3, B5XDG3, B9EQI5, B5DG30, B5XBY4, B5DGI9, C0HAB6, B9EM16, Q9W6K6), carp myofibrillar proteins (P53479, P83750, Q6TKP4, Q6TKP5, Q7T2J3, Q90339, Q2HX57, Q2HX56, Q5NTZ3, Q90337, O42352, Q2HX58, Q90331, Q90332, Q90333, Q9I892, Q90335), carp sarcoplasmic proteins (P09227, P02618, Q8UUS3, Q8UUS2, P02204, Q2LC33, Q8UW95, Q8UW92, O13135, Q8UW94, Q8UW93, O13140), and other carp proteins (P02016, P02139, Q7ZZH6, Q7T276).

### Methods

**Fig 1:**
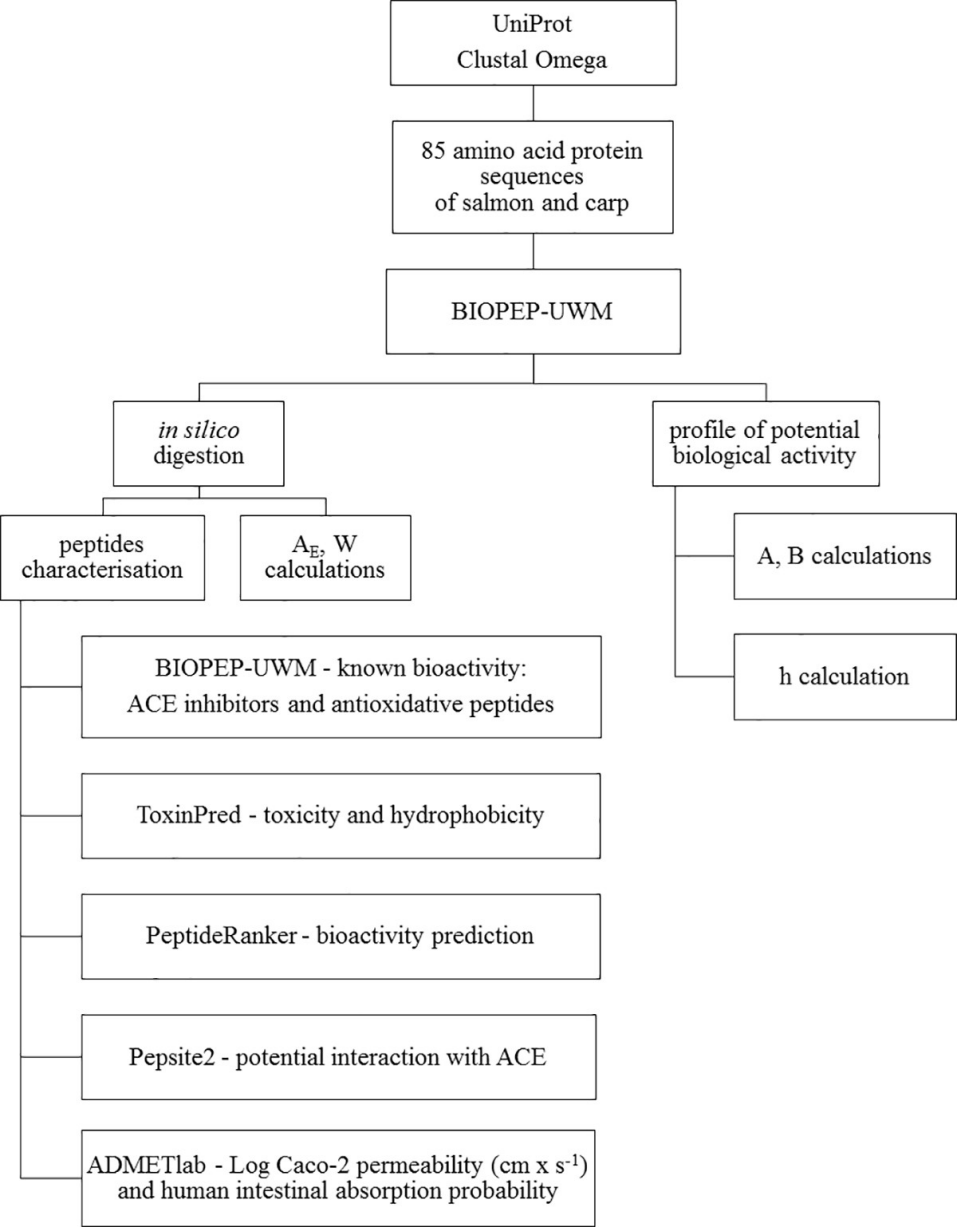
Workflow of conducted research.

The workflow of the conducted analyses is shown in Fig 1.

#### Profiles of the potential biological activity, and A, B, and h parameters

The selected amino acid sequences of salmon and carp proteins acquired from the UniProt database (Table 1) were analyzed using tools available in the BIOPEP-UWM database (provider: University of Warmia and Mazury in Olsztyn, Poland; http://www.uwm.edu.pl/biochemia/index.php) [17].

**Table 1:** Summary of data obtained from determinations of the potential biological activity profiles of selected proteins of salmon (*Salmo salar*) and carp (*Cyprinus carpio*).

		Salmon						Carp					
		Myofibrillar proteins		Sarcoplasmic proteins		The other proteins		Myofibrillar proteins		Sarcoplasmic proteins		The other proteins	
		max	min	max	min	max	min	max	min	max	min	max	min
**Bioactive peptides**	**a**	313 myosin (A8WCK1)	48 myosin (B9ELW1)	88 myoglobin (B9ENY2)	52 parvalbumin (B9EPT7)	1943 collagen (A7KE05)	93 hemoglobin (B5X8L0)	933 MHC (Q5NTZ3)	32 meromyosin (Q90335)	106 myoglobin (P02204)	60 parvalbumin (P09227, Q8UUS2, Q8UUS3)	401 HSP (Q7ZZH6)	99 hemoglobin (P02016)
	**A**	0.6373 MLC (B5DGT1)	0.3551 myosin fr. (C0PU27)	0.6667 myoglobin (B5X603)	0.4771 parvalbumin (B9EPT7)	1.4014 collagen (C1K2L7)	0.4498 HSP (B5DGI9)	0.6321 MLC (Q90332)	0.4503 MLC (Q90333)	0.7273 α-globin (O13135)	0.5139 α-globin (Q8UW92)	0.7063 hemoglobin (P02139)	0.4768 HSP (Q7ZZH6)
**ACE inhibitory peptides**	**a**	199 myosin (A8WCK1)	27 myosin (B9ELW1)	50 myoglobin (B9ENY2)	33 parvalbumin (B9EPT7)	969 collagen (A7KE05)	49 hemoglobin (B5X746)	646 MHC (Q5NTZ3)	23 meromyosin (Q90335)	73 myoglobin (P02204)	42 α-globin (Q8UW92)	286 HSP (Q7ZZH6)	59 hemoglobin (P02016)
	**A**	0.4301 MLC (B5DGT1)	0.2099 myosin fr. (C0PUQ9)	0.4587 parvalbumin (B5DGI8)	0.3028 parvalbumin (B9ENR7)	0.7167 collagen (C0H9S7)	0.278 serum albumin (P21848)	0.4111 actin (Q6TKP5)	0.3271 MHC (Q2HX56, Q2HX58)	0.4966 myoglobin (P02204)	0.2917 α-globin (Q8UW92)	0.4336 hemoglobin (P02139)	0.2762 HSP (Q7ZZH6)
	**B [μM** ^**-1**^ **]**	0.0306 myosin (B5XCI5)	0.003 myosin fr. (C0PU27)	0.0073 parvalbumin (Q91482)	0.0024 myoglobin (B9ENY2)	0.3231 collagen (C0H9S7)	0.0055 HSP (B5DG30)	0.0592 MHC (Q2HX57)	0.0029 MLC (Q90333)	0.034 β-globin (O13140)	0.0053 parvalbumin (Q8UUS3)	0.0338 hemoglobin (P02139)	0.0055 HSP (Q7T276)
**Antioxidant peptides**	**a**	39 myosin (A8WCK1)	3 MLC (B5DGT2)	7 parvalbumin (Q91482)	2 parvalbumin (B5X6D1)	53 collagen (A7KE05)	1 HSP (B5XBY4)	120 MHC (Q2HX56)	2 meromyosin (Q90335)	16 myoglobin (P02204)	3 parvalbumin (P0218, Q8UUS2)	44 HSP (Q7ZZH6)	11 hemoglobin (P02016, P02139)
	**A**	0.0837 myosin (B5XCI5)	0.0186 MLC (B5DGT2)	0.0645 myoglobin (B5X603)	0.0182 parvalbumin (B5X6D1)	0.0699 hemoglobin (B5X746)	0.0048 HSP (B5XBY4)	0.0619 MHC (Q2HX56)	0.0323 meromyosin (Q90335)	0.1088 myoglobin (P02204)	0.0275 parvalbumin (Q8UUS2)	0.0769 hemoglobin (P02016, P02139)	0.0391 HSP (Q7T276)
**Σx**	**a**	238 myosin (A8WCK1)	40 myosin fr. (C0PUQ9)	63 myoglobin (B5DGI8)	37 parvalbumin (B9ENR7, B9EPT7)	1022 collagen (A7KE05)	58 hemoglobin (B5X8L0)	754 MHC (Q2HX56, Q2HX57)	25 meromyosin (Q90335)	89 myoglobin (P02204)	48 parvalbumin (P09227, Q8UUS3)	330 HSP (Q7ZZH6)	70 hemoglobin (P02016)
	**A**	0.4568 myosin (A8WCK1)	0.2469 myosin fr. (C0PUQ9)	0.5138 parvalbumin (B5DGI8)	0.3394 parvalbumin (B9ENR7, B9EPT7)	0.7465 collagen (C0H9S7)	0.3206 HSP (B5DGI9)	0.4536 actin (Q6TKP5)	0.3709 MLC (Q90333)	0.6054 myoglobin (P02204)	0.3611 α-globin (Q8UW92)	0.5105 hemoglobin (P02139)	0.3924 HSP (Q7ZZH6)

Where: a—the number of fragments with a given activity in a protein sequence, A—the frequency of bioactive fragments occurrence in a protein sequence, B—the potential antihypertensive activity of a protein, and Σx—the sum of peptidic ACE inhibitors and antioxidants; HSP—heat shock protein, MLC—myosin light chain, MHC—myosin heavy chain, and fr.–fragment of protein.

The presence of ACE-inhibitory peptides and antioxidant peptides in the chosen proteins were predicted using a database function "Profiles of potential biological activity". According to Minkiewicz et al. [17], the profile of the potential biological activity of a given protein is defined as the type and the location of peptide with a specific activity in a protein chain. These calculations were done using the test protocol accessible *via* the BIOPEP-UWM database consisting of the following steps (available *via* correspondent database bars): (i) BIOPEP-UWM; (ii) Proteins; (iii) Analysis; (iv) Profiles of potential biological activity; (v) Select activity; (vi) Protein database (selection of tested protein).

The frequency of occurrence of ACE inhibitory peptides/antioxidant peptides in a selected protein sequence (*A*) was determined using the following formula:

$$A=\frac{a}{N}$$ (1)

where: a–the number of fragments with an ACE inhibitory activity/antioxidant activity in a protein sequence, N–the number of amino acid residues of a protein.

These calculations were done using the test protocol accessible *via* the BIOPEP-UWM database consisting of the following steps: (i) BIOPEP-UWM; (ii) Proteins; (iii) Analysis; (iv) Calculations; (v) Select activity—this option required selecting activity: “ACE inhibitor” or “antioxidative” when developing the toolbar; (vi) For your sequence–this option required pasting the protein sequence; (vii) Report—the result of the calculation of A value will appear.

Potential ACE inhibitory activity of selected proteins (B) was determined using the following formula:

$$B_{i}=\frac{\sum_{i=1}^{k}\frac{a_{i}}{{IC}_{50i}}}{N}$$ 2)

where: ai−the number of repetitions of the i-th ACE inhibitory fragment in a protein sequence, IC50i –the concentration of the i-th ACE inhibitory peptide corresponding to its half-maximal inhibition [μM], and k–the number of different fragments with a given activity.

These calculations were done using the test protocol accessible *via* the BIOPEP-UWM database consisting of the same steps as for parameter A. IC_50_ values for experimentally validated ACE inhibitory peptides downloaded by database curators were extracted from BIOPEP-UWM database.

The selected proteins were divided into two families, i.e. representing a good or poor potential source of ACE inhibitory and antioxidant peptides. The division was performed according to procedure proposed by Dziuba and Darewicz [23].

The protein sequences were classified depending on the frequency of occurrence of fragments exhibiting ACE inhibitory/antioxidant activities. Proteins selected for further analyses, i.e., simulated *in silico* digestion, included a good source of ACE inhibitory/antioxidant peptides, i.e. those for which the value of parameter A computed for both mentioned activities was higher or equal to the value of parameter h [23]:

$$h=A_{max}-A_{min}$$ (3)

The selected salmon and carp proteins were analyzed separately in groups of myofibrillar, sarcoplasmic, and other proteins.

#### *In silico* proteolysis

The proteolysis simulation tool available in the BIOPEP-UWM database [17], was employed to determine the possibility of release of ACE inhibitory/antioxidant peptides using human digestive enzymes: pepsin [EC 3.4.23.1], trypsin [EC 3.4.21.4], and chymotrypsin [EC 3.4.21.1] The option called “Enzyme(s) action” was used for simulated human-like gastrointestinal digestion. The following steps were applied: (i) BIOPEP-UWM; (ii) Proteins; (iii) Analysis; (iv) Enzyme action; (v) For your sequence (paste the protein sequence); (vi) Select the enzymes; (vii) View the report with the results.

The obtained *in silico* digests were characterized by determining the frequency (A_E_) and the relative frequency (W) of the release of fragments with ACE inhibitory or antioxidant activity from the salmon and carp proteins by the above-mentioned digestive enzymes according to the following formulas [24]:

$$A_{E}=\frac{d}{N}$$ (4)

where: d–the number of peptides with ACE inhibitory/antioxidant activity released by human digestive enzymes, N–the number of amino acid residues of a protein.


$$W=\frac{A_{E}}{A}$$ (5)

#### Structure-based peptides analysis

The peptides released during hydrolysis were analyzed as follows. Their toxicity and hydrophobicity were scored in ToxinPred (provider: Indraprastha Institute of Information Technology, India; http://crdd.osdd.net/raghava/toxinpred/) using a virtual scanning method (VSM) Swiss-Prot based and an SVM threshold of 0.0 http://www.imtech.res.in/raghava/toxinpred/multi_submit.php[25]. The overall bioactivity of the *in silico* released peptides was estimated using tools available in PeptideRanker (provider: University College Dublin, Ireland; http://distilldeep.ucd.ie/PeptideRanker/) [26]. The PeptideRanker tool can order a set of peptides and, based on their function-structure models, assign the scores within the range of 0–1. The highest score is indicative of the most active peptides, and the lowest one–of the least active ones. Peptides for which the PeptideRanker Score was higher than 0.5 were analyzed using Pepsite2 (provider: University of Heidelberg, Germany; http://pepsite2.russelllab.org.) [27], which allows computing the potential interaction (*p*-value) between the peptide and ACE (1O8A, from PDB—Protein Data Bank; provider: Research Collaboratory for Structural Bioinformatics; http://www.rcsb.org/pdb/). In addition, this tool was also used to predict the potential binding sites of ACE with the analyzed peptides. Due to the importance of ACE activity, only the amino acid residues of the active site (H383, H387, E411, and E384) and stabilizing residues (Q281, H353, F457, K511, H513, Y520 and Y523) of ACE were taken into account [28].

Moreover, the human intestinal absorption and predicted Caco-2 permeability of peptides were calculated using the ADMETlab platform (provider: Central South University, China; http://admet.scbdd.com/) [29]. SMILES (The Simplified Molecular Input Line Entry Specification) strings of peptides, including ionization, were used as the input (S6 Table). Amino acid sequences of peptides were converted into SMILES strings using the “SMILES” application available at the BIOPEP-UWM website [17]. Characteristic for neutral pH ionization were created using a molecule editor Marvin JS (ChemAxon, Hungary), available at the SwissTargetPrediction website (provider: Swiss Institute of Bioinformatics, Switzerland; http://www.swisstargetprediction.ch/) [30].

## Results

### Profiles of the potential biological activity and A, B, and h parameters of selected salmon and carp proteins

Peptidic fragments featuring the ACE inhibitory activity and antioxidant peptides were identified in the salmon and carp protein sequences chosen for analyses from UniProt [21] based on the results obtained using the Clustal Omega program and the length of their amino acid sequences (S2 and S3 Tables). The sequences of ACE inhibitory peptides identified in salmon proteins contained peptides composed of 2 to 10 amino acid residues, whereas those identified among carp proteins were built of 2 to 9 amino acid residues. In turn, the sequences of antioxidant peptides identified in salmon proteins contained peptides constituted by 2 to 4 amino acid residues, whereas the respective peptides identified in carp proteins were built of 2 to 17 amino acid residues.

Table 1 summarizes data obtained from determinations of the potential biological activity profiles of selected salmon and carp proteins and results of their bioevaluation. Among the analyzed salmon proteins, collagen (A7KE05) turned out to be potentially the richest source of peptidic ACE inhibitors– 969 fragments, and peptides exhibiting antioxidant activity– 53.

Among the myofibrillar and sarcoplasmic proteins of salmon, myosin (A8WCK1) was potentially the richest source of ACE inhibitors and antioxidant fragments, i.e., 199 and 39 peptides, respectively.

Parameter A (frequency of ACE inhibitors/antioxidant peptides occurrence) enables a quick comparison of the potential of proteins as a source of biopeptides according to the following rule: a protein with a higher A value calculated is a better source of peptides with a given activity. In the case of salmon proteins, the maximal frequency of ACE inhibitors occurrence was determined for collagen (C0H9S7) –0.7167, and the lowest one–for a myosin fragment (C0PUQ9) –0.2099. In turn, the maximal frequency of antioxidant fragments occurrence was determined for myosin (B5XCI5) and reached 0.0831, whereas the lowest one was found for the heat shock protein (B5XBY4) and reached 0.0048.

Parameter B enables searching for proteins representing sources of peptides with the potentially highest value of a given bioactivity (ACE inhibitory in our study) according to the following rule: higher ACE inhibitory activity is predicted for a protein with a higher B value calculated. Among the salmon proteins, the highest potential ACE inhibitory activity (parameter B) was found for collagen (C0H9S7)– 0.3231 μM^-1^, and the lowest one for myoglobin (B9ENY2)– 0.0024 μM^-1^. Considering the myofibrillar and sarcoplasmic proteins, the highest value of this parameter was noted for myosin (B5XCI5)– 0.0306 μM^-1^.

When comparing results obtained for the groups of myofibrillar, sarcoplasmic, and other proteins of salmon, it can be concluded that collagen turned out to be the best potential source of the searched peptides–ACE inhibitors and antioxidant peptides. The potential frequency of occurrence and the potential biological activity were the highest for collagen among all analyzed proteins.

Among the analyzed carp proteins, myosin heavy chain (MHC) from the group of myofibrillar proteins appeared to be potentially the best source of peptidic ACE inhibitors– 646 fragments and antioxidant peptides– 120 fragments, respectively chains Q5NTZ3 and Q2HX57.

Considering carp proteins, the highest frequency of occurrence of the ACE inhibitory and antioxidant peptides was demonstrated for myoglobin (P02204) from the group of sarcoplasmic proteins, i.e. 0.4966 and 0.1088, respectively. The highest potential ACE inhibitory activity (parameter B) was found for the myosin heavy chain—MHC (Q2HX57)– 0.0592 μM^-1^, and the lowest one for the myosin light chain—MLC (Q90332)– 0.0029 μM^-1^.

To sum up, it can be concluded that the myofibrillar proteins of carp are a better source of ACE inhibitory peptides featuring higher potential ACE inhibitory activity and of antioxidant peptides than the sarcoplasmic and other proteins; however, the sarcoplasmic proteins are characterized by a higher frequency of occurrence of bioactive peptides.

The value of parameter h was computed for all selected salmon and carp proteins (Table 2) assuming that an increase in A value enhances the probability of release of bioactive peptides. The proteins, in the case of which the frequency of occurrence of peptides with ACE inhibitory activity and antioxidant activity, and both types of peptides in total (A_Σx_) was higher than the value of parameter h were classified as good sources of peptides with a given activity.

**Table 2:** Values of parameter h computed for selected salmon and carp proteins.

Fish species	Proteins	h _ACEi_	h _antioxidant_	h _Σx_
**Salmon**	myofibrillar	0.2209	0.0651	0.2099
	sarcoplasmic	0.1560	0.0463	0.1743
	other	0.4388	0.0651	0.4259
**Carp**	myofibrillar	0.0840	0.0297	0.0827
	sarcoplasmic	0.2049	0.0813	0.2443
	other	0.0935	0.0378	0.1181

parameter h—difference between the highest and lowest value of the frequency of occurrence (A) of fragments exhibiting ACE inhibitory, antioxidant, and both (Σx) activities.

### *In silico* simulated human-like gastrointestinal digestion of selected salmon and carp proteins

Results of simulated pepsin+trypsin+chymotrypsin proteolysis of the selected amino acid sequences of salmon and carp proteins are presented in S4 Table, which shows the maximum and minimum numbers of released peptides with ACE inhibitory and antioxidant activities (d), as well as potential frequencies (A_E_) and relative frequencies (W) of their release due to the simulated digestion. It can be assumed that the higher the A_E_ value, the higher the number of antioxidant peptides/peptides with ACE inhibitory activity released by the enzyme. In turn, a high value of parameter W suggests that a given enzyme contributes to the release of a large percentage of fragments with an antioxidant/ACE inhibitory activity from protein.

The simulated proteolysis of salmon and carp proteins led to the release of fragments exhibiting the activity of ACE inhibitors (S4 Table).

Among the analyzed salmon proteins, the highest number of potentially ACE inhibitory peptides was released from the amino acid sequence of collagen (A7KE05), i.e., 89 peptides. In turn, the poorest sources of ACE inhibitors potentially released during hydrolysis turned out to be myosin (B9ELW1), with 2 potentially released bioactive fragments.

In turn, the simulated *in silico* digestion of carp proteins allowed for the potential release of 108 ACE inhibitory peptides from the myosin heavy chain. In contrast, the lowest number of ACE inhibitors (i.e., 2) was released from the myosin light chain (Q9I892).

The highest frequencies of ACE inhibitors released (A_E_) were determined for salmon myosin light chain (B5DGT2) and carp myoglobin hydrolysate (Q2LC33)– 0.745 and 0.0748 respectively. The relative frequency of ACE inhibitors release (W) determined after simulated digestion reached the highest value for the myosin light chains of salmon and carp, i.e. 0.2090 and 0.1841 values, respectively.

The fragments exhibiting the antioxidant activity were released by the simulated hydrolysis from 77 amino acid sequences of salmon and carp proteins. Again, the myofibrillar proteins turned out to be a richer source of the desired BPs than the sarcoplasmic proteins. The simulated digestion released potentially the highest number of antioxidant peptides (i.e., 13) from the myosin heavy chain (Q2HX57) of carp (S4 Table). In turn, the sarcoplasmic proteins achieved higher values of BPs release frequency. The highest potential frequency of the release of peptides with the antioxidant activity (A_E_) was demonstrated for the carp α-globin (Q8UW95), whereas the highest relative frequency of these peptides (W)–for salmon myosin (B5XFD6) and carp α-globin (Q8UW95).

Sequences of dipeptides (S5 Table) followed by sequences of tripeptides prevailed among the ACE-inhibiting peptides potentially released from salmon and carp proteins during *in silico* digestion. A longer fragment of the ACE inhibitor–a pentapeptide MNPPK–was identified only in a hydrolysate of carp myofibrillar proteins. In turn, only di- and tripeptides were identified among the peptides with antioxidant activity potentially released from salmon and carp proteins during *in silico* hydrolysis. The myofibrillar proteins of salmon and carp turned out to be richer sources of ACE inhibitors and antioxidant peptides compared to the sarcoplasmic proteins and to the group of other proteins. All sequences of peptidic ACE inhibitors and antioxidant peptides potentially released from proteins of salmon and carp were not potentially toxic, whereas hydrophobicity of some of them justified their solubility in water.

### Structure-based peptide analysis

Table 3 provides sequences of potentially the most active ACE inhibitors and antioxidant peptides (selected according to the PeptideRanker [26] score < 0.5) released *in silico* from the sequences of selected proteins of salmon and carp through the action of pepsin, trypsin, and chymotrypsin. According to Mooney et al. [31], it is presumed that a peptide with PeptideRanker Score >0.5 will show bioactivity in experimental conditions. Among the 63 sequences obtained after the simulated digestion of salmon and carp proteins, 30 were considered potentially bioactive peptides. The peptides with MF, CF, GF, GW, PW, and RF sequences, characterized by the highest PeptideRanker tool score (score ˃ 0.99), were potentially the most active. Although the PeptideRanker tool allows arranging a set of peptides from the most active to the least active one, it fails to predict the actual biological activity of peptides. Therefore, the structure-activity relationship of peptides with ACE inhibitory activity was established in the next stage of the study by determining Pepsite2 p-value [27]. The p-values of the interactions among ACE/ACE binding sites and peptides are presented in Table 3. The lowest p-value was found for peptide PPK (2.60E-06), followed by peptides PR (6.66E-06), MNPPK (1.00E-05), and GPA (1.78E-05). All of them were significantly interacting with ACE (p<0.05). Table 3 also shows potential binding sites of ACE. All 30 peptides can interact with one or more stabilizing residues, i.e., Q281, H353, A354, K511, H513, Y520, and Y523 [32], or zinc ligands of ACE consisting of H383, H387, E411, and a water molecule [33].

**Table 3:** The predicted amino acid sequences and properties of the most potentially active ACE inhibitory peptides and antioxidant (*) peptides matching the salmon and carp protein sequences after *in silico*-simulated human digestion.

No	Amino acid sequence	BIOPEP-UWM ID	Precursor protein^1^	PeptideRanker score	Pepsite2 *p*-value	Potential binding^2^ sites of ACE	Human intestinal absorption probability	Log Caco-2 Permeability (cm x s^-1^)	Bioavailability *in vitro*/ *in vivo*
1	PPK	7545	Carp/M; Salmon/M	0.61	2.60E-06	Q281, H353, H383, E384, E411, K511, H513, Y520, Y523	0.244	-5.890	SHR [34]
2	PR	3537	Carp/M, O; Salmon/M, O	0.79	6.66E-06	Q281, H535, F457, H513, Y520, Y523	0.342	-6.352	SHR [35]
3	PW*	8190	Carp/M, S, O; Salmon/M, O	0.99	8.76E-06	Q281, H353, F457, H513, Y520, Y523	0.467	-5.651	-
4	MNPPK	7571	Carp/M	0.66	1.00E-05	Q281, H353, A354, H387, E411, K511, H513, Y520, Y523	0.250	-6.655	SHR [34]
5	GPA	3342	Carp/M; Salmon/M, O	0.73	1.78E-05	H353, F457, H513, Y520, Y523	0.283	-5.441	-
6	PL	7513	Carp/M, S, O; Salmon/M, S, O	0.81	2.61E-05	Q281, H353, A354, F457, K511, H513, Y520, Y523	0.444	-5.191	Caco-2 [36]
7	PHL*	8029	Salmon/O	0.61	3.25E-05	Q281, H353, F457, K511, H513, Y520, Y523	0.341	-6.207	-
8	GPL	7506	Salmon/ O	0.89	5.44E-05	F457, K511, H513, Y520, Y523	0.325	-5.366	-
9	VPW*	8188	Salmon/M	0.88	5.82E-05	Q281, H353, F457, Y520, Y523	0.374	-5.880	-
10	PGL	7507	Salmon/M, O	0.86	7.40E-05	E411, F457, K511, H513, Y520, Y523	0.309	-5.643	-
11	GHF	7637	Carp/M; Salmon/O	0.95	2.05E-04	H353, E411, H513, Y520, Y523	0.343	-6.459	-
12	GF	7591	Carp/M, S, O; Salmon/M, S, O	0.99	4.58E-04	Q281, H353, A354, Y520, Y523	0.482	-5.354	SHR [37,38]
13	GR	7603	Carp/M, S, O; Salmon/M, O	0.77	5.87E-04	Q281, F457, K511, H513, Y520, Y523	0.335	-6.292	-
14	GA	7598	Carp/M, S, O; Salmon/M, S, O	0.52	5.99E-04	Q281, H353, F457, H513, Y520, Y523	0.430	-5.327	-
15	MF	3385	Carp/M; Salmon/M	1.00	7.22E-04	Q281, H353, F457, H513, Y520, Y523	0.497	-5.405	-
16	GW	7579	Carp/M; Salmon/M, O	0.99	7.71E-04	Q281, F457, K511, H513, Y520, Y523	0.525	-5.735	-
17	RF	3489	Salmon/O	0.99	8.19E-04	Q281, H353, E384, F457, K511, Y520, Y523	0.407	-6.293	SHR [39]
18	NF	7683	Carp/M, S, O; Salmon/M, S, O	0.94	1.06E-03	Q281, H353, E384, K511, H513, Y520, Y523	0.372	-5.907	SHR [37]
19	TF	8185	Carp/M, S, O; Salmon/M, S, O	0.83	1.14E-03	Q281, H353, F457, K511, H513, Y520, Y523	0.310	-5.781	Caco-2 [40]
20	CF	7751	Carp/ M, O; Salmon/ M, O	1.00	1.16E-03	Q281, H353, K511, H513, Y520, Y523	0.480	-5.696	-
21	SF	7685	Carp/M, S, O; Salmon/M, S, O	0.95	1.62E-03	Q281, H353, K511, H513, Y520, Y523	0.281	-5.818	SHR [37]
22	VW and VW*	3486 / 8461	Carp/O; Salmon/O	0.80	2.03E-03	Q281, H353, E411, F457, K511, H513, Y520, Y523	0.575	-5.754	SHR [39,41]
23	IF	7593	Carp/M, O; Salmon/M, S, O	0.95	2.09E-03	H353, E411, F457, Y520, Y523	0.594	-5.355	-
24	IW	7544	Carp/M; Salmon/M, O	0.94	2.09E-03	Q281, H353, E411, H513, Y520, Y523	0.680	-5.794	Human [42] SHR [41]
25	VF	3384	Carp/M, O; Salmon/M, S, O	0.82	3.02E-03	Q281, H353, K511, H513, Y520, Y523	0.543	-5.372	-
26	GY	3532	Carp/M; Salmon/M, O	0.74	3.32E-03	Q281, H353, F457, H513, Y520, Y523	0.325	-5.809	SHR [37,38]
27	RL	3257	Salmon/O	0.63	3.75E-03	Q281, H353, H383, K511, H513, Y520, Y523	0.398	-5.643	-
28	GL	7599	Carp/M, S, O; Salmon/M, S, O	0.81	3.88E-03	Q281, F457, K511, H513, Y520, Y523	0.432	-5.283	-
29	MY and MY*	3388 / 8090	Carp/M; Salmon/M	0.84	5.89E-03	Q281, H353, K511, H513, Y520, Y523	0.350	-5.852	SHR [43]
30	SDF*	7869	Salmon/M	0.77	1.21E-02	Q281, H353, E411, K511, H513, Y520, Y523	0.274	-6.096	-

^1^ Groups of protein: M—myofibrillar, S—sarcoplasmic, O–other

^**2**^ the stabilizing residue or the residue of the active site.

The remaining properties of the peptides calculated using the ADMETlab program [29] are shown in Table 3. The predicted human intestinal absorption ranged from 0.244 for PPK peptide to 0.68 for IW. In turn, the predicted permeability of Caco-2 peptides was low and ranged from -6.655 for the MNPPK sequence to -5.191 for PL. Table 3 also provides information about the models used in the previously published bioavailability studies of the analyzed peptides.

## Discussion

Several studies have been conducted to demonstrate that food ingredients can modulate functions of, e.g., vascular, endocrinal, hormonal, nervous, or digestive systems [44,45]. By doing so, they can be a source of compounds determining the proper functioning of a human body and regulating its psychophysical activity.

Growing attention has recently been observed in research analyzing the behavior of food and its constituents in the human gastrointestinal tract. A load of structured information on the essential physiological functions of food ingredients, including peptides and proteins, can be found in biological and chemical databases [46]. The analysis of biologically active peptides using computer tools is called the *in silico* approach. It is one of the preferred research strategies for food-derived peptides [20,47]. These computer-aided research models enable searching for peptides in various databases and/or using simulation tools to analyze the biological activity and physicochemical properties of peptides derived from food proteins [20]. In the present study, the *in silico* analyses were conducted with the following bioinformatic tools–databases and computer apps: BIOPEP-UWM, UniProtKB, Clustal Omega, PeptideRanker, Pepsite2, and ToxinPred. They allowed evaluating salmon and carp proteins as potential sources of ACE-inhibiting and antioxidant peptides and characterizing products of their *in silico* simulation of human-like gastrointestinal digestion regarding their applicability in the prevention of cardiovascular diseases.

In many studies, the BIOPEP-UWM database of bioactive peptides and proteins of food was recommended as a source of information on food-derived BPs and offered a complex tool for their identification and characterization [17]. It comprises four integrated databases of: proteins, bioactive peptides, sensory peptides and amino acids, and allergenic proteins with their epitopes. It also provides an algorithm that enables evaluating the potential biological activity of proteins according to quantitative criteria.

### Profiles of the potential biological activity, and A and B parameters of selected salmon and carp proteins

The following parameters were designed to aid the assessment of proteins as precursors of bioactive peptides: the profile of the potential bioactivity of a protein, the frequency of occurrence of fragments featuring an ACE inhibitory or antioxidant activity (A), and potential ACE inhibitory activity of protein fragments (B). Determinations of bioactivity profiles and parameter A were used while developing a protein classification system involving their division into families, depending on the prevailing activity type [23]. Parameter A was used to develop profiles of the potential antihypertensive activity of hen myosin [48] and cod proteins [49]. In turn, Carrera et al. [50] discussed the profiles of the potential bioactivity of peptide fragments identified among products of hydrolysis of sarcoplasmic proteins of demersal and pelagic fish species. The BIOPEP-UWM database was also employed to develop potential bioactivity profiles of catla collagen fragments [51], whereas Cho and Kim [52] used sequences of peptides identified in mackerel muscle protein hydrolysates as search queries in BIOPEP-UWM. Finally, Altınelataman et al. [53] employed the BIOPEP-UWM database to determine the potential bioactivity profiles of peptide fragments identified in protein hydrolysates obtained from European seabass and gilthead seabream.

The sequences of the analyzed salmon and carp proteins had from 62 (carp meromyosin, Q90335) to 1938 (carp myosin heavy chain, Q2HX56) amino acid residues. While searching for bioactive sequences, a known rule has been confirmed that the longer the protein sequence is, the higher is the possibility of finding a bioactive fragment. This rule was also confirmed in our previous studies [23,54] and in the research by Yi et al. [55], who showed that, among the analyzed proteins, the collagen subunit protein was the largest group with the greater matching of the antihypertensive peptides. It was also suggested that the number of bioactive fragments in collagen was linked with high numbers of G and P residues, which are amino acid residues frequently occurring in bioactive fragments, including ACE inhibitors and antioxidative peptides. All proteins chosen for the *in silico* analysis in our study turned out to be potential sources of ACE-inhibiting and antioxidant peptides with a predominant proportion of P, G, and F. Results obtained allow concluding that proteins of salmon and carp can be a source of bioactive peptides exhibiting the activity of ACE inhibitors and, to a lesser extent, a source of peptides with antioxidant activity. Similar findings were reported by Carrera et al. [50], who established that the proteome of fish sarcoplasmic proteins was predominated by sequences of antihypertensive peptides, and by Yi et al. [55], who demonstrated antihypertensive peptides in sequences of 17 fish species. It seems that the best source of BPs should be proteins found in large amounts in the muscle cells of salmon and carp, which are also a potentially rich source of bioactive peptides, i.e., collagen, myofibrillar, and sarcoplasmic proteins. Similar conclusions were formulated by Minkiewicz et al. [24] from their *in silico* study of bovine proteins, who demonstrated that the best potential sources of ACE inhibitory and antioxidant peptides among beef muscle proteins turned out to be collagen, hemoglobin, myoglobin, myosin, and actin.

### *In silico* simulated human-like gastrointestinal digestion of selected salmon and carp proteins

The computer analysis of 52 amino acid sequences of salmon proteins and 33 sequences of carp proteins showed they contained bonds susceptible to the activity of the tested endopeptidases, i.e., pepsin, trypsin, and chymotrypsin. While analyzing the release of bioactive peptides, consideration should be given to, i.e., bioactive fragments abundance in the intact protein, the specificity of the enzymes used, and hydrolysis conditions (temperature, pH, enzyme-substrate ratio). The specificity of enzymes’ effects on food proteins can depend on modifications of the protein structure triggered by changes in environmental parameters [56]. Furthermore, interactions of proteins with other compounds of raw materials and food products can partly inhibit or accelerate the proteolysis. Unfortunately, only a few types of specific computer software enable considering and programming conditions of protein hydrolysis [57]. For this reason, it is likely that results obtained under *in silico* conditions will only in part be consistent with results of analytical experiments.

Considering the *in silico* conditions, theoretically, the richer the profile of a protein’s potential bioactivity, the higher the probability of BPs release upon the use of endopeptidases [15]. Salmon collagen and carp myosin revealed both the richest profile of the potential bioactivity of both ACE-inhibiting and antioxidant peptides as well as the highest number of these peptides released after *in silico* hydrolysis with the aforementioned enzymes.

A comparative analysis of the results of *in silico* studies conducted for the protein sequences of salmon and carp demonstrated that the number of bioactive sequences and the frequency of their occurrence were higher in the case of carp than salmon.

The analysis of our study results allows concluding that the pepsin+trypsin+chymotrypsin hydrolysis promotes the release of peptidic ACE inhibitors from the amino acid sequences of salmon and carp proteins. The hydrolysis resulted in the release of a significantly higher number of fragments with the ACE-inhibiting than with the antioxidant activity. It is notable that most of the ACE inhibitors present in the sequences of the analyzed proteins were built of two amino acids, while the antioxidant peptides–from three amino acids. The probability of a diamino acid fragment release is higher than that of the triamino acid fragment, especially that the *in silico* research assumes a hydrolysis efficiency of 100%.

Among the salmon proteins, collagen was indicated as the protein with the highest frequency of occurrence of ACE-inhibiting fragments and with the highest number of released peptides exhibiting this activity. Among the myofibrillar and sarcoplasmic proteins, myosin was the best source of the desired fragments, presumably due to the length of its polypeptidic chain. In was also characterized by the highest frequency of the peptidic ACE inhibitors release. This was due to a higher frequency of occurrence of bonds susceptible to the action of pepsin, trypsin, and chymotrypsin in the analyzed proteins of salmon as well as to the shorter length of the polypeptidic chain of myosin than collagen. The shorter the protein sequence and the higher the number of potentially released BPs, the higher the value of the A_E_ parameter. Furthermore, the highest values of the relative frequency of ACE inhibitors release (parameter W) from myofibrillar and sarcoplasmic proteins of salmon were computed also for myosin. The value of parameter W indicates the probability of the release of fragments with a given activity from a protein with a known frequency of occurrence of such fragments. The higher the value of parameter W, the greater the probability and the “effectiveness” of the release of bioactive fragments from sequences of the intact protein.

Among the carp proteins, myosin showed the highest frequency of occurrence and the highest number of released peptidic ACE inhibitors. However, the highest frequency of release of these antihypertensive fragments was demonstrated for myoglobin sequences, whereas the highest relative frequency of their release–for myosin sequences.

Among the carp proteins, the highest frequency of occurrence of the antioxidant fragments was demonstrated for the heat shock protein; however, myosin turned out to be the richest source of peptides featuring this activity after hydrolysis. The highest frequency of release and the highest relative frequency of release of the antioxidant peptides was reported for globin hydrolysates.

The analysis of myofibrillar and sarcoplasmic proteins of salmon and carp demonstrated that, for most of the proteins tested, the values of parameter W were higher for these exhibiting the antioxidant than the ACE-inhibiting activity. This was indicative of the higher probability of release of fragments featuring the antioxidant activity from amino acid sequences of the analyzed proteins despite the lower frequency of their occurrence and the lower frequency of their release from polypeptidic chains. These results prove a greater match of the specificity of enzymes used for the *in silico* hydrolysis to the type of amino acid residues present at the C-terminus in the sequences of the antioxidant peptides than of the antihypertensive peptides. These observations are in accordance with results concerning myofibrillar proteins–actin and myosin, which were identified by Vercruysse et al. [58] as good sources of antioxidant peptides.

To sum up, the analysis of myofibrillar and sarcoplasmic proteins of salmon and carp showed that the highest numbers of peptides with ACE inhibiting and antioxidant activities were released from collagen and myosin, which obtained the highest values of A_E_ (given enzymes can release bioactive fragments) and W (given enzymes contribute to the release of a high percentage of fragments with a given activity) parameters. The high values of A_E_ and W may suggest that the given protein is a good source of biopeptides and that the applied enzymes are useful in releasing BPs. Similar results were obtained under *in silico* conditions by Minkiewicz et al. [24] for beef collagen and elastin as well as by Yunhai et al [55] for salmon collagen. Results of predictions made under *in silico* conditions can be confirmed under *in vitro* conditions. Scientists from various research centers, who identified sequences of antihypertensive peptides in fish protein hydrolysates, demonstrated that the majority of them were derived from myofibrillar proteins and collagen. For example, Enari et al. [59] identified 20 di- and tripeptides with the ACE inhibitory activity in a papain hydrolysate of pink salmon (*Oncorhynchus gorbuscha*) muscle. In turn, Gu et al. [60] identified 11 sequences of peptidic ACE inhibitors in a hydrolysate of a collagen extract from salmon skin.

### Structure-based peptide analysis

Studies on the relationship between the structure and the ACE inhibitory and antioxidant activity have shown that the inhibitory activity and antioxidant capacity of peptides is closely related to their structural features, such as molecular weight and hydrophobicity [61]. Most of the peptides selected in our study for *in silico* analyses were hydrophobic (S5 Table). Wu and Aluko [62] highlighted the influence of physicochemical features of amino acids, such as hydrophobicity and electronic properties (ionization state), on the biological activity of peptides of food origin. Moreover, the bioactivity of peptides is influenced by the type and number of amino acid residues as well as their position in the sequence [7]. The amino acid sequences of ACE-inhibiting and antioxidant peptides released in our study after simulated digestion with pepsin, trypsin, and chymotrypsin were predominated by the following amino acid residues (listed acc. to a descending contribution in sequences): P, G, F, W, R, and L. It is well-known that the specific amino acid composition of peptides determines their bioactivity [63]. The some regularities can be observed between the presence of the specific amino acid(s)and the function of a whole peptide sequence. The results of analytical and chemometric experiments allowed establishing certain structural features of ACE inhibitors [7]. The peptides that contain proline, which has a cyclic structure, can introduce kinks to the structure of ACE that can closely interact with other stabilizing residues [64]. Many previous works have mentioned P next to R, H, Y, F, and W among the amino acids affecting the enzymatic activity inhibition [65]. Hydrophobic amino acids, particularly these with aliphatic chains, like G, I, L, and V, are typical of the N-terminus of peptidic ACE inhibitors [7]. In our study, amino acids with aliphatic chains, such as G and L, and amino acids with cyclic or aromatic rings, like P and F, were predominantly present in peptides released after hydrolysis (Table 3).

The antioxidant activity of food peptides is also directly linked with the amino acid composition, structure and position of the chain [66,67]. Carboxyl and amino groups in the side chains of the acidic (G, Q, D,N) and basic (K, H, R) amino acids are known to play an important role in chelating metal ions [68]. The main antioxidative mechanism of peptides with free radical quenching activity was reported to be associated with the presence of amino acids, such as P, A, V and L [69]. Many antioxidative peptides identified include hydrophobic amino acid residues V or L at the N-terminus end and P, H or Y in the sequences [70]. What is more, some amino acids, such as aromatic residues, H, M, C, P, A, or G, are believed to enhance radical scavenging activities of antioxidant peptides [69,70]. In our study, amino acids with aliphatic chains, such as V, and amino acids with cyclic or aromatic rings, like P and W, were predominantly present in peptides released after hydrolysis (Table 3).

Computational tools can aid in elaborating the interaction mechanisms of BPs with receptors from the binding sites and binding types between the receptor and ligands. Twenty one peptides with the PeptideRanker Score ˃ 0.5 selected for the analysis with PepSite2 were dipeptides, eight were tripeptides, and one was pentapeptide (MNPPK). This peptide was identified as an ACE inhibitor from porcine myosin heavy chain (IC_50_ = 945.5 μM) [71] and chicken myosin after simulated gastric digestion [72]. The PPK peptide is known as an ACE inhibitor from porcine myosin (weaker than MNPPK), but it also showed antithrombotic activity as a fragment of bovine κ-casein [73]. The RL peptide is, in turn, the ACE inhibitory peptide from whey protein. It also showed the antidiabetic activity as the dipeptidyl peptidase IV (DPP-IV) inhibitor.

Some of the analyzed peptides had previously been identified as ACE inhibitors derived from fish proteins. GPL, PGL, and PL peptides were identified in Alaskan pollack skin gelatin hydrolysates, where the first one was most active (IC_50_ = 2.65μM), and the last one was the weakest inhibitor (IC_50_ = 337.32μM) [74]. In turn, VF (IC_50_ = 9.20μM), MF (IC_50_ = 45μM), and MY (IC_50_ = 193μM) peptides were identified in sardine muscle alkaline protease hydrolysate [75]. VF and MF peptides were described as a competitive ACE inhibitors, whereas MY as an noncompetitive one. All these peptides were also identified in tryptic amaranth glutelin digests [76]. Peptide CF is an ACE inhibitor from shark meat released by protease SM98011 from *Bacillus sp*. (IC_50_ = 1.96μM). Chum salmon muscle hydrolyzed by thermolysin was the source of VW and IW peptides [41]. Both of them were also identified in plant proteins, amaranth, and cereals. In turn, GPA peptide, which was known as a synthetic ACE inhibitor, was *in silico* identified in chum salmon collagen, bovine meat collagen and myosin, and canola proteins also as a DPP-IV inhibitor [77]. Nine of the analyzed peptides presented in Table 3 were of plant origin. PR (IC_50_ = 4.1μM), RF (IC_50_ = 93μM), and GY (IC_50_ = 210μM) peptides were observed in sake and amaranth protein hydrolysates [35,76]. NF (IC_50_ = 46.3μM) and SF (IC_50_ = 130.2μM) peptide were obtained from a garlic protein hydrolysate [37]. The TF peptide was identified in wheat and rapeseed protein hydrolysates. Peptides PW and VPW are the antioxidant peptides from *in vitro* digest of buckwheat proteins [78], whereas SDF is an antioxidant peptide from okara protein Protease N hydrolysate [79]. The remaining analyzed peptides were from peptide libraries analyzed for ACE inhibitory activity (excluding the PHL which is from antioxidant library) and, apart from the GW peptide, were characterized by low activity (IC_50_ > 900μM). Most of the analyzed peptides with ACE inhibitory activity (mainly dipeptides) showed at least 2 activities simultaneously. Most often, it was the activity of DPP-IV inhibitor, rarely DPP-III inhibitor, renin inhibitor, and some were inhibitors of other enzymes.

Computer-based tools based on the chemical similarity can be used to predict biological targets that interact with BPs [80]. The identification of a biological target of bioactive peptides selected in our study was aimed at ACE. The structure-based relationship of selected peptides and ACE was established using Pepsite2. Potential interactions between ACE and peptides were computed, and potential binding sites were identified. The 3-D structure of this glycoprotein shows that it is a zinc metallopeptidase [7]. Zinc ligands of ACE are composed of H383, H387, E411, and a water molecule which is bound to E384. In turn, the primary stabilizing residues are Q281, H353, A354, K511, H513, Y520, and Y523. We established that all studied peptides were significantly interacting with the stabilizing and active site residues of ACE. Peptide PPK, recorded with the lowest *p*-value, interacted with both stabilizing and active site residues. The lowest *p*-value determined for the first top 3 ACE inhibitory peptides, i.e., PPK, PR, and MNPPK, indicates the significance of proline residues. The cyclic structure of proline has been proved responsible for active interactions between ACE and other residues [7]. Worthy of notice is the theoretically confirmed possibility of antioxidant peptides to interact with stabilizing and active site residues of ACE, with the highest potential interaction between the PW peptide and ACE.

The ADMET strategy is largely related to absorption, distribution, metabolism, excretion properties, and toxicities of new substances, mainly drug candidates. It covers the kinetic issues determining whether a molecule will get to the target protein in the body, and how long it will stay in the bloodstream. The analyses of ADMET properties are routinely carried out at the early stage of drug and drug-like discovery. It is, therefore, reasonable to use this method in the analysis of peptides, which can then be used as components of functional foods or supplements. The ADMET properties of peptides were interpreted based on the criteria presented and described on the website of the ADMETlab program [29]. Predicted Caco-2 permeability of peptides was low because it did not exceed −5.28 (according to the program criteria, the optimal logarithm of permeability should exceed −5.15). Caco-2 monolayers are recommended as models for simulating the absorption of compounds from digestive tracts [81]. However, it should be remembered that the Caco-2 cell line is a cancerous line from colon and may not fully correspond to the conditions in the digestive system of a healthy person. That is why, the potential human intestinal absorption (HIA) was also calculates. Most of the analyzed peptides—25 out of the 30, revealed high predicted intestinal absorption probability (>0.3). The highest value of predicted absorption were calculated for IW, IF, VW, VF, and GW peptides. However, the most interesting results were obtained with the PW peptide. It had PeptideRanker Score = 0.99, one of the highest *p*-value (8.76E-0.6) and relatively high human intestinal absorption probability (0.467). The bioactivity of the PW peptide was evaluated *in vitro* [78], while the bioavailability of the IW and VW peptides was previously proven in *in vitro* studies using spontaneously hypertensive rats (SHR) [39,41]. The predicted absorption probability of most of the peptides analyzed and low toxicity of all peptides should be deemed their advantage.

Relatively few studies investigating the bioavailability of bioactive peptides have used *in vitro* cell models, human, rat, or pig models. However, studies in humans, rats (including spontaneously hypertensive rats, SHR), and pigs have provided evidence that certain BPs are absorbed *in vivo*. The putative mechanisms of biopeptide bioavailability were well described by Xu et al. [82]. Some scientists have demonstrated that di- and tripeptides can be absorbed intact into the human circulatory system *via* the intestinal peptide transporter PEPT1 [83,84]. The peptide IW (Table 3) was characterized by the highest human intestinal absorption probability (0680). It should be noted that, among the tested peptides, only IW was previously analyzed using the human model. It is a competitive and selective inhibitor of human ACE, which decreased the ACE activity in plasma by 32 ± 8% following 50 mg oral administration [42]. Kaiser et al. study [42] showed that the tryptophan-containing dipeptides IW and WL were resistant to proteolytic digestion and were readily absorbed in the gastrointestinal tract after oral administration.

The SHR model is the most commonly used model for testing the bioavailability of ACE inhibitors, either directly or indirectly through blood pressure testing. For example, the ACE activity of peptide MY, a dipeptide derived from sardine muscle with the non-competitive inhibition mode [43], was proved using the SHR model [85]. In addition, this model proved successful in demonstrating *in vivo* the antihypertensive activities of peptides MNPPK (myopentapeptides A) and PPK from the thermolysin hydrolysate of porcine muscle myosin [34]. Both peptides reduced systolic blood pressure (SBP) of the spontaneously hypertensive rats 6 h after administration, and PPK also after 24 h. Peptides GY and GF were a part of sea bream scale hydrolysates obtained by commercially available alkaline protease from *Bacillus* sp., which significantly decreased the blood pressure of SHR upon administration at 300 mg/kg of body weight per 1 d [38]. Systolic blood pressure of "young" SHR decreased significantly after oral administration of RF and VW that were isolated from the hydrolysate of sake lee and a peptide fraction of sake [39]. Whereas, when the PR peptide was orally administered to SHR, it caused no significant reduction in their SBP [35]. The upstream chum salmon defatted muscle proteins were hydrolyzed with thermolysin A. As a part of this hydrolysate among others antihypertensive peptides VW and IW were identified [41]. Suetsuna observed that a single dose (200 mg/kg) of various dipeptides from *Allium sativum* L. caused a maximal decrease in SBP at different times after administration, including among others: after 1 hour for NF, GF, and SF; and after 4 hours for GY [37]. In the cited study, oral administration of the analyzed dipeptides showed blood pressure-reducing activity qualitatively similar to that of captopril.

## Conclusions

The results obtained allow concluding that the *in silico* methods used in the present study can be applied to screen sequences of selected salmon and carp proteins to enable the identification of fragments with the ACE inhibitory and antioxidant activity and to establish the possibility of their release *via* human-like simulated digestion with pepsin, trypsin, and chymotrypsin. The amino acid sequences of salmon and carp proteins can be potential sources of peptides exhibiting both angiotensin I-converting enzyme inhibitory activity and antioxidant activity. The highest number of bioactive peptides featuring these activities was potentially released after *in silico* digestion from amino acid sequences of salmon collagen as well as from salmon and carp myosin. Our approach also takes account of bioinformatic techniques of data mining to analyze structure-activity of di-, tri-, and pentapeptides derived from salmon and carp proteins. Considering the *in silico* methods, worthy of attention is the limited possibility of programming the computer simulation of proteolysis that would entail complicated conditions like, e.g., these in the human gastrointestinal tract. For instance, the bioinformatic tools of the BIOPEP-UWM database intended for hydrolysis simulation, take account of the cutting sequence and identification sequences but not the kinetic factors of the enzymatic reactions. Given these limitations, one should keep in mind, that results obtained with the computer-aided methods can only partly correspond to the results of experimental analyses. Nevertheless, it can be stated that, the combined methods and tools of BPs computer research have partially overcome the limitations of traditional research methods. The effectiveness and possibility of biopeptides release *via in silico* tools are determined by building as large as possible collection of protein and peptide sequences in databases (meaning, continuous curation of databases), completing information about as many as possible BPs of the known IC_50_ value, and replenishment of the data concerning the specificity of the action of enzymes used for *in silico* hydrolysis/digestion.

To sum up, the *in silico* methods, becoming more popular among scientists who work on bioactive peptides of foods origin, can be recommended for the screening and preselection of protein sequences to predict the potential bioactivity of their fragments and to search for possibilities of releasing bioactive peptides from precursor sequences. Computational studies enable minimizing the number of experiments involved to determine the effect of the peptide sequence on its biological function. Moreover, *in silico* experiments are relatively easy and less costly to carry out, and do not require reagent and sample preparation. Consistently, bioinformatic methods and tools play an increasingly important role in cutting-edge science and can support analyses of proteins as potential precursors of health-promoting peptides.

## Supporting information

S1 Table The salmon and carp protein sequences chosen for analyses from UniProt, their mass and the length of amino acid sequences. (DOCX)

S2 Table Peptide sequences—ACE inhibitors and antioxidants, identified in the selected salmon (*Salmo salar*) proteins as a result of determining the profiles of biological activity. (DOCX)

S3 Table Peptide sequences—ACE inhibitors and antioxidants, identified in the selected carp (*Cyprinus carpio*) proteins as a result of determining the profiles of biological activity. (DOCX)

S4 Table The results of *in silico* digestion of salmon (*Salmo salar*) and carp (*Cyprinus carpio*) proteins. d—number (maximum and minimum) of potential bioactive peptides predicted to be released per protein molecule, A_E_−frequency, and W—relative frequency of the release of fragments with ACE-inhibitory activity and antioxidant peptides after pepsin, trypsin, and chymotrypsin *in silico* hydrolysis.
(DOCX)

S5 Table The predicted amino acid sequences and properties of ACE-inhibiting and antioxidant peptides matching the salmon (*Salmo salar*) and carp (*Cyprinus carpio*) protein sequences after *in silico* simulated human-like digestion. (DOCX)

S6 Table InChiKey and SMILES strings and structures of peptides with ionized acidic and basic groups released from the salmon and carp protein sequences after *in silico* simulated human-like digestion. (DOCX)
