# Supplementary material for: Properties of peptides released from salmon and carp via simulated human-like gastrointestinal digestion described applying quantitative parameters
Source: PLoS One. 2021 Aug 10;16(8):e0255969. doi: 10.1371/journal.pone.0255969 (PMC8354434; doi:10.1371/journal.pone.0255969)
Supplement: S1 Table — (DOCX) [file pone.0255969.s001.docx]

**S1 Table. The salmon (*Salmo salar*) and carp (*Cyprinus carpio*) protein sequences chosen for analyses from UniProt, their mass and the length of amino acid sequences.**

| **No** | **ID (UniProt)** | **Name of protein (UniProt)** | **Mass (kDa)** | **Amino acid chain length** |
| --- | --- | --- | --- | --- |
| **Salmon (*Salmo salar*) myofibrillar proteins** | | | | |
| 1 | Q78BU2 | Actin alpha 1-1 (Fast myotomal muscle actin) | 41,932 | 377 |
| 2 | B5XFZ3 | Actin, adductor muscle | 41,584 | 376 |
| 3 | B5DG40 | Fast myotomal muscle action 2 | 41,918 | 377 |
| 4 | B5DH12 | Myosin light chain 1-1 | 21,001 | 193 |
| 5 | B5DH13 | Myosin light chain 1-2 | 21,017 | 186 |
| 6 | B5DGT2 | Myosin light chain 3, skeletal muscle isoform | 17,313 | 161 |
| 7 | B9EMP7 | Myosin light polypeptide 4 | 22,658 | 205 |
| 8 | B5XFD6 | Myosin light polypeptide 4 | 22,272 | 198 |
| 9 | B5XFN3 | Myosin light polypeptide 6B | 23,506 | 211 |
| 10 | Q7ZZN0 | Myosin regulatory light chain 2 | 18,996 | 170 |
| 11 | B5X1K8 | Myosin regulatory light chain 2, smooth muscle isoform | 19,818 | 173 |
| 12 | B9ENW2 | Myosin regulatory light chain 2, smooth muscle isoform | 19,228 | 170 |
| 13 | B5XE45 | Myosin regulatory light chain 2, ventricular/cardiac muscle isoform | 19,094 | 168 |
| 14 | B5X5V3 | Myosin regulatory light chain 2B, cardiac muscle isoform | 18,965 | 168 |
| 15 | B5DGT1 | Myosin, light polypeptide 3-1 | 21,001 | 193 |
| 16 | A8WCK1 | Myosin 1 (Fragment) | 55,716 | 521 |
| 17 | C0PU27 | Myosin-11 (Fragment) | 28,305 | 245 |
| 18 | B5XCI5 | Myosin-7 | 23,298 | 203 |
| 19 | C0PU50 | Myosin-9 (Fragment) | 60,187 | 515 |
| 20 | B9ELW1 | Myosin-Ie | 8,969 | 79 |
| 21 | C0PU83 | Myosin-If (Fragment) | 34,714 | 313 |
| 22 | C0PUQ9 | Myosin-IXb (Fragment) | 18,335 | 162 |
| 23 | C0PUQ3 | Myosin-VI (Fragment) | 29,792 | 257 |
| 24 | Q2HXU3 | Slow myosin heavy chain 1 (Fragment) | 25,68 | 228 |
| **Salmon sarcoplasmic proteins** | | | | |
| 25 | B5DGI8 | Parvalbumin 2 | 11,787 | 109 |
| 26 | C0HAT9 | Parvalbumin alpha | 11,893 | 109 |
| 27 | Q91482 | Parvalbumin beta 1 (Major allergen Sal s 1) | 11,889 | 109 |
| 28 | Q91483 | Parvalbumin beta 2 (Major allergen Sal s 1) | 11,383 | 108 |
| 29 | B5X6D1 | Parvalbumin, thymic | 12,075 | 110 |
| 30 | B9ENR7 | Parvalbumin, thymic CPV3 | 12,054 | 109 |
| 31 | B9EPT7 | Parvalbumin, thymic CPV3 | 11,931 | 109 |
| 32 | B5X603 | Myoglobin | 10,09 | 93 |
| 33 | B9ENY2 | Myoglobin | 15,714 | 147 |
| **Salmon other proteins** | | | | |
| 34 | C0H9S7 | Collagen alpha-1XIII chain | 63,312 | 639 |
| 35 | B5X659 | Collagen triple helix repeat-containing protein 1 | 25,115 | 229 |
| 36 | C1K2L7 | Collagen type XI alpha1 short isoform | 58,59 | 588 |
| 37 | A7KE05 | Collagen Type XI Alpha2 (Collagen type XI) | 161,948 | 1590 |
| 38 | C0H805 | Hemoglobin subunit alpha | 25,392 | 235 |
| 39 | B5X746 | Hemoglobin subunit alpha-4 | 15,894 | 143 |
| 40 | B5X8L0 | Hemoglobin subunit beta | 16,181 | 148 |
| 41 | C0H744 | Hemoglobin subunit beta-1 | 15,998 | 147 |
| 42 | P21848 | Serum albumin 1 | 67,151 | 608 |
| 43 | C0HBF1 | 60 kDa heat shock protein, mitochondrial | 61,083 | 577 |
| 44 | B5X4Z3 | Heat shock 70 kDa protein | 70,834 | 644 |
| 45 | B5XDG3 | Heat shock protein 30 | 25,263 | 220 |
| 46 | B9EQI5 | Heat shock protein 30 | 26,019 | 240 |
| 47 | B5DG30 | Heat shock protein 70 isoform 3 | 71,03 | 651 |
| 48 | B5XBY4 | Heat shock protein beta-1 | 23,347 | 208 |
| 49 | B5DGI9 | Heat shock protein beta-11 (Hsp 30-like) | 23,838 | 209 |
| 50 | C0HAB6 | Heat shock protein HSP 90-alpha | 84,64 | 734 |
| 51 | B9EM16 | Heat shock protein Hsp-16.48/Hsp-16.49 | 26,505 | 231 |
| 52 | Q9W6K6 | Heat shock protein hsp90 beta | 83,318 | 722 |
| **Carp (*Cyprinus carpio*) myofibrillar proteins** | | | | |
| 1 | P53479 | Actin-alpha, skeletal muscle | 41,959 | 377 |
| 2 | P83750 | Actin-beta | 41,753 | 375 |
| 3 | Q6TKP4 | Skeletal muscle actin mutant | 41,975 | 377 |
| 4 | Q6TKP5 | Skeletal muscle alpha-actin | 41,929 | 377 |
| 5 | Q7T2J3 | Skeletal muscle actin (Fragment) | 41,961 | 377 |
| 6 | Q90339 | Myosin heavy chain | 221,601 | 1937 |
| 7 | Q2HX57 | Myosin heavy chain embryonic type 2 | 221,895 | 1935 |
| 8 | Q2HX56 | Myosin heavy chain embryonic type 3 | 222,543 | 1938 |
| 9 | Q5NTZ3 | Myosin heavy chain | 220,988 | 1931 |
| 10 | Q90337 | Myosin heavy chain | 221,093 | 1933 |
| 11 | O42352 | Myosin heavy chain | 221,163 | 1931 |
| 12 | Q2HX58 | Myosin heavy chain embryonic type 1 | 221,683 | 1932 |
| 13 | Q90331 | Fast skeletal myosin light chain 1a | 21,128 | 193 |
| 14 | Q90332 | Fast skeletal myosin light chain 1b | 21,134 | 193 |
| 15 | Q90333 | Fast skeletal myosin light chain 3 | 16,805 | 151 |
| 16 | Q9I892 | Myosin regulatory light chain | 18,896 | 169 |
| 17 | Q90335 | Light meromyosin (Fragment) | 6,941 | 62 |
| **Carp sarcoplasmic proteins** | | | | |
| 18 | P09227 | Parvalbumin-alpha | 11,451 | 108 |
| 19 | P02618 | Parvalbumin-beta | 11,436 | 108 |
| 20 | Q8UUS3 | Parvalbumin, cyp c 1.01 | 11,504 | 109 |
| 21 | Q8UUS2 | Parvalbumin, cyp c 1.02 | 11,569 | 109 |
| 22 | P02204 | Myoglobin | 15,776 | 147 |
| 23 | Q2LC33 | Myoglobin isoform 2 | 16,174 | 147 |
| 24 | Q8UW95 | Alpha globin type-2 | 15,8 | 143 |
| 25 | Q8UW92 | Alpha globin type-2 | 15,757 | 144 |
| 26 | O13135 | Alpha globin type-2 | 15,431 | 143 |
| 27 | Q8UW94 | Alpha globin type-2 | 16,706 | 147 |
| 28 | Q8UW93 | Beta globin type-3 | 16,375 | 147 |
| 29 | O13140 | Beta-globin | 16,343 | 148 |
| **Carp other proteins** | | | | |
| 30 | P02016 | Hemoglobin subunit alpha (Hemoglobin alpha chain) (Alpha-globin) | 15,447 | 143 |
| 31 | P02139 | Hemoglobin subunit beta-A/B (Hemoglobin beta-A/B chain) | 16,262 | 143 |
| 32 | Q7ZZH6 | Heat shock protein 4 | 94,463 | 841 |
| 33 | Q7T276 | Muscle-specific heat shock protein Hsc70-1 (Fragment) | 70,362 | 639 |
