# Supplementary material for: Properties of peptides released from salmon and carp via simulated human-like gastrointestinal digestion described applying quantitative parameters
Source: PLoS One. 2021 Aug 10;16(8):e0255969. doi: 10.1371/journal.pone.0255969 (PMC8354434; doi:10.1371/journal.pone.0255969)
Supplement: S2 Table — (DOCX) [file pone.0255969.s002.docx]

S2 Table. Peptide sequences - ACE inhibitors and antioxidants, identified in the selected salmon (*Salmo salar*) proteins as a result of determining the profiles of biological activity.

|  | **ACE inhibitory peptides** | | | | | | | | | | | | | | | **Antioxidant peptides** | | | | | |
| --- | --- | --- | --- | --- | --- | --- | --- | --- | --- | --- | --- | --- | --- | --- | --- | --- | --- | --- | --- | --- | --- |
| **myofibrillar proteins** | AA  AAP  AF  AFP  AG  AGSS  AH  AI  AKK  ALPHA  AP  AR  AW  AY  CF  DA  DG | DLP  EA  EG  EI  EK  EV  EY  FAP  FG  FGK  FP  FQP  FR  FY  GA  GD  GE | | GF  GG GH  GI  GK  GKP  GL  GLP  GM  GP  GPA  GPP  GPV  GQ  GR  GRP  GS | GT  GV  GW  GY  GYALPHA  HG  HK  HL  HP  HP  HY  IA  IAE  IAK  IAP  IE  IF | | ILP  IG  IKP  IKY  IP  IPA  IQY  IR  IRA  IRP  IVGRPRHQG  IVY  IW  IWH  IWHHT  IY  KA | | KE  KF  KG  KL  KP KR  KW  KY  KYY  LA  LAA  LAP  LF  LG  LGP  LKA  LKL | LKP  LLF  LN  LNP  LPG  LQ  LRP  LSP  LVL  LVR  LW  LY  ME  MF  MG  MY  MYPGIA | | NF  NG  NK  NKL  NPP  NY  PAP PG  PGL  PH  PL  PP  PPK  PQ  PQR  PR  PT | QG  QK  RA  RF  RL  RP  RR  RW RY  SF  SG  SY  TE  TF  TG  TQ  TVY | VAA  VAP  VAV  VAY  VE  VF  VFPS  VG  VK  VKAGF  VP  VR  VTVNPYKWLP  VW  VWIG  VY  WG | YA  YALPHA  YG  YK  YL  YP  YQY  YVP | ADF  AH  AY  EAK  EL  FIKK  HH  HIH  HL  IKK  IQY  IR  IY  KD | KP  KYY  LH  LHD LHE  LHF  LHR  LK  LKP  LWA  LWR  LY  MY  PEL | PHA  PHD  PHG  PHI  PW  PWI  PWN  RHQ  RHT  RHV  RW  SDF  TFE  TY | | | VKV  VPW  VY  VYY  WHH  WNIP  WY  YQY  YTY  YVL  YYI  YYS |
| **sarcoplasmic proteins** | AA  AF  AG  AH  AI  AP | AR  DA  DG  EA  EI  EK | EV  FFL  FG  FP  FY  GA | GD  GE  GF  GG  GH  GI | GK  GL  GM  GP  GPV  GQ | GR GV  GW  GY  HG HK | | HL  HP  IA  IE  IF  IG | IP  KA KE  KF  KG  KL | LA  LAA LF  LG  LKA  LKL | LN  LQ LVL  ME  MG  NF | NK  NY PG  PL  PP  RA | RF  RL RP  SF  SG  TE | TF  TG  VAA  VE  VF  VG | VK  VP  WG  YL | AH  EL  FKK  HL | | IKK  KD  KVI  LH | | | LHL  LK  TY  VKV |
| **other proteins** | AA  AAP  AF  AG  AGSS  AH  AI  AKK  AP  AR  AW  AY  CF  DA | DG  DLP  EA EG  EI  EK  EV  EY  FAL  FAP  FG  FP  FR  FY | GA  GD  GE  GEP  GF  GG GH  GHF  GI  GK  GKP  GL  GLP  GM | GP  GPA  GPL  GPP  GPV  GQ  GR  GS  GT GV  GW  GY  GYK HG | HK  HL  HLL  HP  HY  IA  IAE  IAK  IE  IEP  IF  IFG  IG  ILP | IP  IPA  IPP  IQP  IR  IRA  IVY  IW  IY  IYP  KA  KE  KF  KFY | | KG  KL  KP  KR  KW  KY  KYY  LA  LAA  LAP  LF  LG  LGP  LKA | LKL  LLF  LLP  LN  LNP  LPG  LPP  LQ  LQQ  LRY  LSP  LVE  LVL  LVR | LW  LY  LYP  ME  MF  MG  MKG MKY  MNP  MY  NF  NG  NK  NKL | NY  PAP  PG  PGL  PH  PL  PLG  PLP  PLW  PP PQ  PR  PSY  PT | QG  QK  RA  RF  RIY  RL  RP  RR  RW  RY  SF  SG  SY TAP | TE  TF  TG  TQ  VAA  VAP  VAV  VAY  VE  VF  VG  VIY  VK  VLP | VP  VR  VRP  VSP  VW  VY  VYP  WG  YA  YG  YGL  YK  YL  YNK | YP  YPR  YW | ADF  AH  AY  DYY  EAK  EL  FKK  GAH GGE  HDH  HH  HL  IKL | IR  IY  IYY KAI  KD  KP  KVI  KYY  LARL  LH  LHA  LHD  LHE | LHF  LHI  LHM  LHQ  LHV LK  LWG  LWT  LWV  LY  MY  PEL  PHG | PHI  PHL  PHN  PHS  PHV  PW PWI  PWR  PWT  RHA  RHE  RHF  RHH | RHK  RHN  RHY  RW  RWY  RYY  TY VFPW  VKL  VKV  VY  VYY  WY | WYS  YDY  YSY  YVL  YYE  YYI  YYK  YYT |
