# Supplementary material for: Properties of peptides released from salmon and carp via simulated human-like gastrointestinal digestion described applying quantitative parameters
Source: PLoS One. 2021 Aug 10;16(8):e0255969. doi: 10.1371/journal.pone.0255969 (PMC8354434; doi:10.1371/journal.pone.0255969)
Supplement: S3 Table — (DOCX) [file pone.0255969.s003.docx]

S3 Table. Peptide sequences - ACE inhibitors and antioxidants, identified in the selected carp (*Cyprinus carpio*) proteins as a result of determining the profiles of biological activity.

|  | **ACE inhibitory peptides** | | | | | | | | | | | | | | | | **Antioxidant peptides** | | | |
| --- | --- | --- | --- | --- | --- | --- | --- | --- | --- | --- | --- | --- | --- | --- | --- | --- | --- | --- | --- | --- |
| **myofibrillar proteins** | AA  AF  AFP  AG  AH  AI  AIP  AKK  ALPHA  AP  AR  AVP  AW  AY  CF  DA  DG | EA  EG  EI  EK  EV  EW  EY  FG  FGK  FNQ  FP  FQP  FR  FY  GA  GD  GE | GF  GG  GGY  GH  GHF  GI  GK  GKP  GL  GM  GP  GPA  GQ  GR  GRP  GS  GT | GV  GW  GY  GYALPHA  HG  HK  HL  HP  HY  IA  IAE  IAK  IAP  IE  IF  IG  IKP | | | IP  IQY  IR  ITT  ITTNP  IVGRPRHQG  IW  IWH  IWHHT  IY  IYK  KA  KE  KF  KG  KL  KP | | | KR  KW  KY  LA  LAA  LAP  LF  LG  LKA  LKL  LN  LNP  LQ  LQQ  LTF  LW  LY | | | LYP  MDLA  ME  MF  MG  MKG  MNP  MNPPK  MY  MYPGIA  NF  NG  NK  NKL  NPP  NY  PAP | PG  PGL  PH  PL  PP  PPK  PQ  PR  PT  QAFT  QG  QK  RA  RF  RL  RP  RR | RY  SF  SG  SY  TAP  TE  TF  TG  TNP  TQ  TTN  VAA  VAP  VAV  VAY  VE  VF | VFPS  VG  VK  VKAGF  VP  VR  VW  VWIG  VY  YA  YALPHA  YG  YK  YL  YP  YPR  YQY | ADF  AH  AY  EAK  EL  EYY  FIKK  GGE  HH  HL  HPH  IKK  IQY  IR | IY  KAI  KD  KP  LARL  LEQQVDDLEGSLEQEKK  LH  LHA  LHD  LHE  LHG  LHH  LHL  LHR | LHS  LHY  LK  LWA  LWM  LY  MHH  MY  NHH  PEL  PHA  PHF  PHI  PW | RHD  RHI  RHQ  RHV  VKL  VKV  VYY  WHH  YAY  YDY  YQY  YTY  YYI  YYK  YYS |
| **sarcoplasmic proteins** | AA  AF  AG  AH  AI  AP  AR  AW  AY  DA  DG  EA | EG  EI  EK  EV  EW  FG  FP  FR  GA  GD  GE | GF  GG  GH  GI  GK  GL  GM  GP  GPV  GQ  GR | | GS  GT  GV  HG  HK  HL  HP  IA  IE  IF  IG | | IP  IR  IVY  KA  KE  KF  KG  KL  KP  KR  KY | | LA  LF  LG  LGP  LKA  LKL  LKP  LN  LNP  LQ  LQP | | | LSP  LVL  LVVYPWTQR  LW  LY  MF  MG  NF  NK  NY  PG | | PL  PP  PQ  QG  QK  RA  RF  RL  RR  RY  SF | SG  TE  TF  TG  TQ  TVY  VAA  VE  VG  VK  VR | VVYPW  VW  VY  VYP  WG  YA  YG  YGG  YK  YP  YPWTQR | ADF  AH  AY  EL  GGE  HL  IKK | IR  KD  KP  KVI  LARL  LH  LHA | LHG  LHS  LHV  LK  LKP  LWA  LWG | LY  PW  PWT  RYY  TYY  VKV  YVY  YYK |
| **ther proteins** | AA  AF  AFP  AG  AH  AI  AKK  AP  AR  AW  AY  CF  DA | DG  DLP  EA  EG  EI  EK  EV  EW  EY  FG  FGK  FP  FR | FY  GA  GD  GE  GF  GG  GH  GI  GK  GKP  GL  GM  GP | GPV  GQ  GR  GS  GT  GV  GY  HG  HL  IA  IAE  IE  IEP | | IF  IG  IP  IPP  IVY  IW  IY  IYK  KA  KE  KF  KG  KL | | KP  KR  KW  KY  LA  LAA  LAY  LF  LG  LGP  LKL  LLF  LN | | | LNP  LQ  LQQ  LSP  LSPA  LTF  LVE  LVR  LW  LY  LYP  ME  MG | | MY  NF  NG  NK  NY  PAP  PG  PL  PP  PQ  PR  PSY  PT | PYP  QG  QK  RA  RF  RFH  RL  RP  RR  RY  SF  SG  SY | TE  TF  TG  TQ  TVY  VAA  VAV  VAY  VE  VF  VG  VK  VP | AH  AY  AYY  EAK | EL  FKK  GGE  HL  IY  KD  KP | KVI  LH  LHA  LHG  LHV  LK | LWA  LWG  LY  MY  PW  PWT | SDF  TY  VKV  VY  YVY  YYS |
