# Supplementary material for: Properties of peptides released from salmon and carp via simulated human-like gastrointestinal digestion described applying quantitative parameters
Source: PLoS One. 2021 Aug 10;16(8):e0255969. doi: 10.1371/journal.pone.0255969 (PMC8354434; doi:10.1371/journal.pone.0255969)
Supplement: S4 Table — d—number (maximum and minimum) of potential bioactive peptides predicted to be released per protein molecule, AE−frequency, and W—relative frequency of the release of fragments with ACE-inhibitory activity and antioxidant peptides after pepsin, trypsin, and chymotrypsin in silico hydrolysis. (DOCX) [file pone.0255969.s004.docx]

**S4 Table.** **The results of *in silico* digestion of salmon (*Salmo salar*) and carp (*Cyprinus carpio*) proteins,** where: d - number (maximum and minimum) of potential bioactive peptides predicted to be released per protein molecule, A_E_ - frequency and W - relative frequency of the release of fragments with ACE inhibitory activity and antioxidant peptides after pepsin, trypsin and chymotrypsin *in silico* hydrolysis.

|  |  | **Salmon (*Salmo salar*)** | | **Carp (*Cyprinus carpio*)** | |
| --- | --- | --- | --- | --- | --- |
|  | **parameter** | **max** | **min** | **max** | **min** |
| **ACE inhibitory peptides** | d | 89  collagen (A7KE05)  25  myosin  (A8WCK1) | 2  myosin  (B9ELW1) | 108  myosin heavy chain (O42352) | 2  myosin light chain (Q9I892) |
|  | A_E_ | 0.0745  myosin light chain (B5DGT2) | 0.0176  myosin light chain (Q7ZZN0) | 0.0748  myoglobin (Q2LC33) | 0.0339  myosin light chain (Q9I892) |
|  | W | 0.2090  myosin light chain (B5XFD6) | 0.0500  myosin light chain (Q7ZZN0) | 0.1841  myosin light chain (Q90333) | 0.0339  myosin light chain (Q9I892) |
| **Antioxidant peptides** | d | 5  myosin  (A8WCK1) | 0  heat shock protein (B5XBY4, B5XDG3),  hemoglobin (B5X746),  myosin light chain (B5DGT2, B5X1K8),  myosin fragment (C0PU27, C0PU50),  parvalbumin (B5DGI8) | 13  myosin heavy chain (Q2HX57) | 1  α-globin (O13135, Q8UW92),  β-globin (Q8UW93),  hemoglobin (P02016),  myosin light chain (Q90331, Q90332) |
|  | A_E_ | 0.0136  hemoglobin (C0H744) |  | 0.0210  α-globin (Q8UW95) | 0.0031  heat shock protein (Q7T276) |
|  | W | 0.2090  myosin light chain (B5XFD6) |  | 0.2727  α-globin (Q8UW95) | 0.0667  myosin heavy chain (Q2HX56) |
