# Supplementary material for: Properties of peptides released from salmon and carp via simulated human-like gastrointestinal digestion described applying quantitative parameters
Source: PLoS One. 2021 Aug 10;16(8):e0255969. doi: 10.1371/journal.pone.0255969 (PMC8354434; doi:10.1371/journal.pone.0255969)
Supplement: S5 Table — (DOCX) [file pone.0255969.s005.docx]

**S5 Table. The predicted amino acid sequences and properties of ACE-inhibiting and antioxidant peptides matching the salmon (*Salmon salar*) and carp (*Cyprinus carpio*) protein sequences after *in silico* simulated human-like gastrointestinal digestion.**

| **No.** | **Amino acid sequence** | **Fish/Groups of proteins*** | **BIOPEP-UWM ID** | **Activity**** | **PeptideRanker score** | **Prediction of toxicity**** | **Hydrophobicity** |
| --- | --- | --- | --- | --- | --- | --- | --- |
| 1 | MF | Carp/M; Salmon/M | 3385 | ACE | 1.00 | No | 0.43 |
| 2 | CF | Carp/ M, O; Salmon/ M, O | 7751 | ACE | 1.00 | No | 0.33 |
| 3 | GF | Carp/M, S, O; Salmon/M, S, O | 7591 | ACE | 0.99 | No | 0.39 |
| 4 | GW | Carp/M; Salmon/M, O | 7579 | ACE | 0.99 | No | 0.27 |
| 5 | PW | Carp/M, S, O; Salmon/M, O | 8190 | AO | 0.99 | No | 0.15 |
| 6 | RF | Salmon/O | 3489 | ACE | 0.99 | No | -0.57 |
| 7 | IF | Carp/M, O; Salmon/M, S, O | 7593 | ACE | 0.95 | No | 0.67 |
| 8 | SF | Carp/M, S, O; Salmon/M, S, O | 7685 | ACE | 0.95 | No | 0.17 |
| 9 | GHF | Carp/M; Salmon/O | 7637 | ACE | 0.95 | No | 0.12 |
| 10 | IW | Carp/M; Salmon/M, O | 7544 | ACE | 0.94 | No | 0.55 |
| 11 | NF | Carp/M, S, O; Salmon/M, S, O | 7683 | ACE | 0.94 | No | -0.02 |
| 12 | GPL | Salmon/ O | 7506 | ACE | 0.89 | No | 0.21 |
| 13 | VPW | Salmon/M | 8188 | AO | 0.88 | No | 0.28 |
| 14 | PGL | Salmon/M, O | 7507 | ACE | 0.86 | No | 0.21 |
| 15 | MY | Carp/M; Salmon/M | 3388 | ACE | 0.84 | No | 0.14 |
|  | MY | Carp/M; Salmon/M | 8090 | AO | 0.84 | No | 0.14 |
| 16 | TF | Carp/M, S, O; Salmon/M, S, O | 8185 | ACE | 0.83 | No | 0.21 |
| 17 | VF | Carp/M, O; Salmon/M, S, O | 3384 | ACE | 0.82 | No | 0.57 |
| 18 | PL | Carp/M, S, O; Salmon/M, S, O | 7513 | ACE | 0.81 | No | 0.23 |
| 19 | GL | Carp/M, S, O; Salmon/M, S, O | 7599 | ACE | 0.81 | No | 0.35 |
| 20 | VW | Carp/O; Salmon/O | 3486 | ACE | 0.80 | No | 0.46 |
|  | VW | Carp/O; Salmon/O | 8461 | AO | 0.80 | No | 0.46 |
| 21 | PR | Carp/M, O; Salmon/M, O | 3537 | ACE | 0.79 | No | -0.92 |
| 22 | SDF | Salmon/M | 7869 | AO | 0.77 | No | -0.12 |
| 23 | GR | Carp/M, S, O; Salmon/M, O | 7603 | ACE | 0.77 | No | -0.80 |
| 24 | GY | Carp/M; Salmon/M, O | 3532 | ACE | 0.74 | No | 0.09 |
| 25 | GPA | Carp/M; Salmon/M, O | 3342 | ACE | 0.73 | No | 0.11 |
| 26 | MNPPK | Carp/M | 7571 | ACE | 0.66 | No | -0.32 |
| 27 | RL | Salmon/O | 3257 | ACE | 0.63 | No | -0.61 |
| 28 | PPK | Carp/M; Salmon/M | 7545 | ACE | 0.61 | No | -0.41 |
| 29 | PHL | Salmon/O | 8029 | AO | 0.61 | No | 0.02 |
| 30 | GA | Carp/M, S, O; Salmon/M, S, O | 7598 | ACE | 0.52 | No | 0.21 |
| 31 | SG | Salmon/M | 7618 | ACE | 0.41 | No | -0.05 |
| 32 | PQ | Carp/S, O; Salmon/M, O | 7837 | ACE | 0.39 | No | -0.38 |
| 33 | PHA | Carp/M; Salmon/M | 8022 | AO | 0.38 | No | -0.07 |
| 34 | GQ | Carp/M, S, O; Salmon/M, S, O | 7610 | ACE | 0.38 | No | -0.26 |
| 35 | HL | Carp/M, S, O; Salmon/S, O | 7602 | ACE | 0.37 | No | 0.07 |
|  | HL | Carp/M, S, O; Salmon/S, O | 3317 | AO | 0.37 | No | 0.07 |
| 36 | RA | Salmon/O | 7588 | ACE | 0.35 | No | -0.76 |
| 37 | IR | Carp/M; Salmon/M, O | 3258 | ACE | 0.33 | No | -0.52 |
|  | IR | Carp/M; Salmon/M, O | 8215 | AO | 0.33 | No | -0.52 |
| 38 | IY | Carp/M; Salmon/O | 3383 | ACE | 0.32 | No | 0.38 |
|  | IY | Carp/M; Salmon/O | 7873 | AO | 0.32 | No | 0.38 |
| 39 | HY | Carp/M; Salmon/M, O | 3494 | ACE | 0.30 | No | -0.19 |
| 40 | GK | Carp/M, S, O; Salmon/M, S, O | 7611 | ACE | 0.30 | No | -0.47 |
| 41 | GGE | Carp/O; Salmon/ O | 8114 | AO | 0.29 | No | -0.10 |
| 42 | SY | Carp/M, O; Salmon/M, O | 7684 | ACE | 0.26 | No | -0.12 |
| 43 | KL | Salmon/other proteins | 7693 | ACE | 0.23 | No | -0.29 |
| 44 | NY | Carp/M, S, O; Salmon/M, S, O | 7682 | ACE | 0.22 | No | -0.31 |
| 45 | VE | Carp/M, S, O; Salmon/M, S, O | 7829 | ACE | 0.22 | No | -0.04 |
| 46 | ME | Carp/M, O; Salmon/M, S, O | 7839 | ACE | 0.20 | No | -0.18 |
| 47 | TG | Salmon/O | 7621 | ACE | 0.19 | No | -0.01 |
| 48 | IA | Carp/M, S, O; Salmon/M, O | 7562 | ACE | 0.18 | No | 0.49 |
| 49 | DA | Carp/ M, S, O; Salmon/M, S, O | 7606 | ACE | 0.13 | No | -0.23 |
| 50 | VIY | Salmon/O | 7749 | ACE | 0.12 | No | 0.43 |
| 51 | IVY | Carp/S, O; Salmon/O | 7541 | ACE | 0.12 | No | 0.43 |
| 52 | VR | Carp/M, O; Salmon/M, O | 7628 | ACE | 0.11 | No | -0.61 |
| 53 | TY | Carp/M, S, O; Salmon/M, O | 8219 | AO | 0.11 | No | -0.08 |
| 54 | GE | Carp/M, S, O; Salmon/M, S, O | 7615 | ACE | 0.11 | No | -0.23 |
| 55 | VAY | Salmon/M | 3546 | ACE | 0.11 | No | 0.27 |
| 56 | HK | Carp/S; Salmon/M, S, O | 7844 | ACE | 0.10 | No | -0.75 |
| 57 | VY | Carp/M, S, O; Salmon/M, O | 3492 | ACE | 0.10 | No | 0.28 |
|  | VY | Carp/M, S, O; Salmon/M, O | 8224 | AO | 0.10 | No | 0.28 |
| 58 | NK | Carp/M, S, O; Salmon/M, S, O | 7698 | ACE | 0.06 | No | -0.87 |
| 59 | TVY | Carp/S, O; Salmon/M, O | 7498 | ACE | 0.06 | No | 0.13 |
| 60 | IE | Carp/M, S, O; Salmon/M, S, O | 7827 | ACE | 0.05 | No | 0.05 |
| 61 | TQ | Carp/M, S, O; Salmon/M, O | 7834 | ACE | 0.04 | No | -0.43 |
| 62 | VK | Carp/M, S, O; Salmon/M, S, O | 7558 | ACE | 0.03 | No | -0.28 |
| 63 | TE | Carp/M, S, O; Salmon/M, S, O | 7830 | ACE | 0.02 | No | -0.40 |

*Groups of protein: M - myofibrillar, S - sarcoplasmic, O – other.

**Activities: ACE – ACE inhibitory, AO- antioxidant.
