# Supplementary material for: Properties of peptides released from salmon and carp via simulated human-like gastrointestinal digestion described applying quantitative parameters
Source: PLoS One. 2021 Aug 10;16(8):e0255969. doi: 10.1371/journal.pone.0255969 (PMC8354434; doi:10.1371/journal.pone.0255969)
Supplement: S6 Table — (DOCX) [file pone.0255969.s006.docx]

**S6 Table. InChiKey and SMILES strings and structures of peptides with ionized acidic and basic groups released from the salmon (*Salmon salar*) and carp (*Cyprinus carpio*) protein sequences after *in silico* simulated human-like gastrointestinal digestion.**

| **No** | **Amino acid sequence** | **BIOPEP-UWM ID** | **Prekursor protein^1^** | **InChIKey** | **SMILES** |
| --- | --- | --- | --- | --- | --- |
| 1 | PPK | 7545 | Carp/M; Salmon/M | PCWLNNZTBJTZRN-AVGNSLFASA-N | [H][C@@](CCCC[NH3+])(NC(=O)[C@]1([H])CCCN1C(=O)[C@]1([H])CCC[NH2+]1)C([O-])=O |
| 2 | PR | 3537 | Carp/M, O; Salmon/M, O | HMNSRTLZAJHSIK-YUMQZZPRSA-N | [H][C@@](CCCNC(N)=[NH2+])(NC(=O)[C@]1([H])CCC[NH2+]1)C([O-])=O |
| 3 | PW* | 8190 | Carp/M, S, O; Salmon/M, O | UEKYKRQIAQHOOZ-KBPBESRZSA-N | [H][C@]1(CCC[NH2+]1)C(=O)N[C@@H](Cc1c[nH]c2ccccc12)C([O-])=O |
| 4 | MNPPK | 7571 | Carp/M | PWVZDSOAAGBGIA-VMXHOPILSA-N | [H][C@]([NH3+])(CCSC)C(=O)N[C@@]([H])(CC([NH3+])=O)C(=O)N1CCC[C@@]1([H])C(=O)N1CCC[C@@]1([H])C(=O)N[C@@]([H])(CCCC[NH3+])C([O-])=O |
| 5 | GPA | 3342 | Carp/M; Salmon/M, O | GGLIDLCEPDHEJO-BQBZGAKWSA-N | [H][C@@](C)(NC(=O)[C@]1([H])CCCN1C(=O)C[NH3+])C([O-])=O |
| 6 | PL | 7513 | Carp/M, S, O; Salmon/M, S, O | ZKQOUHVVXABNDG-IUCAKERBSA-N | [H][C@@](CC(C)C)(NC(=O)[C@]1([H])CCC[NH2+]1)C([O-])=O |
| 7 | PHL* | 8029 | Salmon/O | STASJMBVVHNWCG-IHRRRGAJSA-N | [H][C@@](CC(C)C)(NC(=O)[C@H](Cc1c[nH]cn1)NC(=O)[C@]1([H])CCC[NH2+]1)C([O-])=O |
| 8 | GPL | 7506 | Salmon/ O | HFPVRZWORNJRRC-UWVGGRQHSA-N | [H][C@@](CC(C)C)(NC(=O)[C@]1([H])CCCN1C(=O)C[NH3+])C([O-])=O |
| 9 | VPW* | 8188 | Salmon/M | NSUUANXHLKKHQB-BZSNNMDCSA-N | [H][C@]([NH3+])(C(C)C)C(=O)N1CCC[C@@]1([H])C(=O)N[C@@H](Cc1c[nH]c2ccccc12)C([O-])=O |
| 10 | PGL | 7507 | Salmon/M, O | FKLSMYYLJHYPHH-UWVGGRQHSA-N | [H][C@@](CC(C)C)(NC(=O)CNC(=O)[C@]1([H])CCC[NH2+]1)C([O-])=O |
| 11 | GHF | 7637 | Carp/M; Salmon/O | HPAIKDPJURGQLN-KBPBESRZSA-N | [H][C@@](Cc1ccccc1)(NC(=O)[C@H](Cc1c[nH]cn1)NC(=O)C[NH3+])C([O-])=O |
| 12 | GF | 7591 | Carp/M, S, O; Salmon/M, S, O | JBCLFWXMTIKCCB-VIFPVBQESA-N | [H][C@@](Cc1ccccc1)(NC(=O)C[NH3+])C([O-])=O |
| 13 | GR | 7603 | Carp/M, S, O; Salmon/M, O | JLXVRFDTDUGQEE-YFKPBYRVSA-N | [H][C@@](CCCNC(N)=[NH2+])(NC(=O)C[NH3+])C([O-])=O |
| 14 | GA | 7598 | Carp/M, S, O; Salmon/M, S, O | VPZXBVLAVMBEQI-VKHMYHEASA-N | [H][C@@](C)(NC(=O)C[NH3+])C([O-])=O |
| 15 | MF | 3385 | Carp/M; Salmon/M | HGCNKOLVKRAVHD-RYUDHWBXSA-N | [H][C@]([NH3+])(CCSC)C(=O)N[C@@]([H])(Cc1ccccc1)C([O-])=O |
| 16 | GW | 7579 | Carp/M; Salmon/M, O | AJHCSUXXECOXOY-NSHDSACASA-N | [NH3+]CC(=O)N[C@@H](Cc1c[nH]c2ccccc12)C([O-])=O |
| 17 | RF | 3489 | Salmon/O | PQBHGSGQZSOLIR-RYUDHWBXSA-N | [H][C@](N)(CCCNC(N)=[NH2+])C(=O)N[C@@]([H])(Cc1ccccc1)C([O-])=O |
| 18 | NF | 7683 | Carp/M, S, O; Salmon/M, S, O | OMSMPWHEGLNQOD-UWVGGRQHSA-N | [H][C@]([NH3+])(CC(N)=O)C(=O)N[C@@]([H])(Cc1ccccc1)C([O-])=O |
| 19 | TF | 8185 | Carp/M, S, O; Salmon/M, S, O | IQHUITKNHOKGFC-MIMYLULJSA-N | [H][C@](C)(O)[C@]([H])([NH3+])C(=O)N[C@@]([H])(Cc1ccccc1)C([O-])=O |
| 20 | CF | 7751 | Carp/ M, O; Salmon/ M, O | XZFYRXDAULDNFX-UWVGGRQHSA-N | [H][C@]([NH3+])(CS)C(=O)N[C@@]([H])(Cc1ccccc1)C([O-])=O |
| 21 | SF | 7685 | Carp/M, S, O; Salmon/M, S, O | PPQRSMGDOHLTBE-UWVGGRQHSA-N | [H][C@]([NH3+])(CO)C(=O)N[C@@]([H])(Cc1ccccc1)C([O-])=O |
| 22 | VW and VW* | 3486 / 8461 | Carp/O; Salmon/O | LZDNBBYBDGBADK-KBPBESRZSA-N | [H][C@]([NH3+])(C(C)C)C(=O)N[C@@H](Cc1c[nH+]c2ccccc12)C([O-])=O |
| 23 | IF | 7593 | Carp/M, O; Salmon/M, S, O | WMDZARSFSMZOQO-DRZSPHRISA-N | [H][C@](C)(CC)[C@]([H])([NH3+])C(=O)N[C@@]([H])(Cc1ccccc1)C([O-])=O |
| 24 | IW | 7544 | Carp/M; Salmon/M, O | BVRPESWOSNFUCJ-LKTVYLICSA-N | N[C@@]([H])([C@]([H])(CC)C)C(=O)N[C@@H](Cc1c[nH]c2c1cccc2)C(=O)O |
| 25 | VF | 3384 | Carp/M, O; Salmon/M, S, O | GJNDXQBALKCYSZ-RYUDHWBXSA-N | [H][C@]([NH3+])(C(C)C)C(=O)N[C@@]([H])(Cc1ccccc1)C([O-])=O |
| 26 | GY | 3532 | Carp/M; Salmon/M, O | XBGGUPMXALFZOT-VIFPVBQESA-N | [H][C@@](Cc1ccc(O)cc1)(NC(=O)C[NH3+])C([O-])=O |
| 27 | RL | 3257 | Salmon/O | WYBVBIHNJWOLCJ-IUCAKERBSA-N | [H][C@]([NH3+])(CCCNC(N)=[NH2+])C(=O)N[C@@]([H])(CC(C)C)C([O-])=O |
| 28 | GL | 7599 | Carp/M, S, O; Salmon/M, S, O | DKEXFJVMVGETOO-LURJTMIESA-N | [H][C@@](CC(C)C)(NC(=O)C[NH3+])C([O-])=O |
| 29 | MY and MY* | 3388 / 8090 | Carp/M; Salmon/M | DKEXFJVMVGETOO-LURJTMIESA-N | [H][C@]([NH3+])(CCSC)C(=O)N[C@@]([H])(Cc1ccc(O)cc1)C([O-])=O |
| 30 | SDF* | 7869 | Salmon/M | OLIJLNWFEQEFDM-SRVKXCTJSA-N | [H][C@](N)(CO)C(=O)N[C@@]([H])(CC([O-])=O)C(=O)N[C@@]([H])(Cc1ccccc1)C([O-])=O |

* antioxidant peptides

^1^Groups of protein: M - myofibrillar, S - sarcoplasmic, O – other.
